# Paravertebral Catheter for Three-Level Injection in Radical Mastectomy: A Randomised Controlled Study

**DOI:** 10.1371/journal.pone.0129539

**Published:** 2015-06-09

**Authors:** Petchara Sundarathiti, Benno von Bormann, Ronnarat Suvikapakornkul, Panuwat Lertsithichai, Vanlapa Arnuntasupakul

**Affiliations:** 1 Department of Anaesthesiology, Ramathibodi Hospital, Mahidol University, Bangkok, Thailand; 2 Department of Anaesthesiology, Siriraj Hospital, Mahidol University, Bangkok, Thailand; 3 Department of Surgery, Ramathibodi Hospital, Mahidol University, Bangkok, Thailand; ACTREC (Advanced Centre for Treatment, Research and Education in Cancer) / Tata Memorial Centre, INDIA

## Abstract

**Introduction:**

Paravertebral block (PVB) is an alternative to general anaesthesia (GA) for breast surgery. However, for extensive surgery multiple punctures are needed increasing the immanent risk of the method. The purpose of this study was to evaluate PVB via catheter and injections at three different levels. Primary outcome was the quality of postoperative analgesia, in particular, the number of patients requiring additional morphine.

**Methods:**

In a randomised single blinded clinical study patients scheduled for breast surgery including axillary approach, were randomly allocated to different anaesthetic techniques, n = 35 each. Patients received either GA with sevoflurane or PVB with catheter at level Th 4. In PVB-patients a 1:2 mixture of bupivacaine 0.5% and lidocaine 2% with adrenaline was injected sequentially 10 ml each at three different levels.

**Results:**

Complication-free catheter insertion was possible in all 35 scheduled patients. The need for postoperative analgesics was higher after GA compared to PVB (22 vs.14 patients); p = 0.056. Postoperative morphine consumption was 1.55 (GA) and 0.26 mg (PVB) respectively (p < 0.001). Visual rating score (VRS) for pain at rest and at movement was higher in GA patients on post anaesthesia care unit (PACU) as well as on the ward at 1 - 6h and 6 - 12h. Readiness for discharge was earlier after PVB (4.96 and 6.52 hours respectively). After GA the incidence and severity of postoperative nausea and vomiting (PONV) was higher, though not significantly. Patients’ satisfaction was comparable in both groups.

**Conclusions:**

Three-level injection PVB via catheter for extensive mastectomy was efficient and well accepted. Using a catheter may enhance safety by avoiding multiple paravertebral punctures when extended spread of analgesia is required.

**Trial Registration:**

www.ClinicalTrial.gov NCT02065947

## Introduction

Benefits suggested for paravertebral block (PVB) alone or combined with general anaesthesia in breast surgery are improving postoperative pain control and pulmonary function, decreasing cancer recurrence and reducing length of hospital stay [1–4]. Failure rates of up to 20% have been reported after single injection PVB [5,6]. For radical mastectomy including axilla revision sufficient analgesia has to be provided for at least nine dermatomes, C6—Th 6 [7]. Attempts have been made to acquire adequate surgical conditions by applying multiple paravertebral injections [8,9]. Considering the potential risks of paravertebral block [10–13] and the stress for the patients a limitation of punctures seems to be preferable. Inserting a paravertebral catheter far enough and in proper position offers the opportunity to apply the anaesthetic agent at different sites with one puncture only, by moving the catheter backwards after each injection. Up to now there is no such study.

The purpose of our study was to evaluate a single-puncture, three-injection technique via catheter as a sole anaesthetic technique in extensive breast surgery comparing it to standard general anaesthesia. First objective was its effect on postoperative analgesic quality regarding number of patients requiring analgesics. Additionally we repeatedly recorded the postoperative pain scores until 24^th^ hour and the total dosage of IV morphine given as a rescue drug. Further objectives were readiness for discharge, incidence of postoperative nausea and vomiting and patients’ satisfaction.

## Methods

This is a prospective, randomized, single-blinded clinical trial. There is currently no similar study running within our department. Approval from the Institutional Review Board (IRB) Ramathiboti Hospital, Mahidol University Bangkok was obtained on October 24^th^ 2013 (No. MURA2013/534; S1 and S2 Files). Date range for patient recruitment and follow-up was October 25^th^, 2013 (1^st^ patient recruited), till April 24^th^, 2014 (last follow-up day of the final patient No 70). Patients had to agree to participate, documented by *written* informed consent; all patients included in this study did so (S3, S4, S5 and S6 Files).

### Patients

Female patients, with ASA (American Society of Anesthesiologists) risk score I—III undergoing unilateral mastectomy surgery with axillary dissection (radical lymph node removal) were enrolled. Exclusion criteria were general infection and local infection at the site of puncture, anatomic deformities of the thoracic spine, coagulation disorders, allergy against local anaesthetics or contrast agents (Iopamiro 300), pregnancy or breast-feeding and BMI ≥ 30 kg/m^2^. Patients were randomly assigned to two groups by using block-of-four method, general anaesthesia (GA) or paravertebral block (PVB).

#### Preoperative preparation, similar for all patients

After arrival at the OR standard monitoring was applied including ECG, non-invasive blood pressure and pulse oximetry. Midazolam 0.05 mg per kilogram bodyweight (kgbw) was given intravenously to achieve mild sedation.

### General anaesthesia (GA)

Anaesthesia was induced intravenously with 1.5–2 mg/kg propofol, fentanyl 1–2 mcg/kg and atracurium to facilitate tracheal intubation, and maintained with sevoflurane in oxygen and air 50:50%. Additional Fentanyl 1–2 mcg/kg was administered on discretion of the anesthesiologist.

### Paravertebral block (PVB)

Paravertebral block was performed by the first author (P.S.) only with the patient in prone position modifying the technique described by Eason and Wyatt [7]. Modification included the position of the patients which was described by Eason and Wyatt either lateral with the side to be blocked uppermost or sitting upright. Similar to them was the direction of the needle during puncture. An 18-gauge Tuohy needle without stylet but with a saline filled syringe attached was inserted perpendicular to the skin at T3–T4 interspace. The needle was advanced ultrasound-guided using transverse view out-of-plane technique and if necessary directed cephalad parallel to the spine until loss of resistance (LOR). An end-hole 20-gauge catheter was advanced 8 cm beyond needlepoint into the paravertebral space, the manoeuvre rated as ‘easy’, ‘difficult’ (change of needle direction, use of saline solution for dilatation), or ‘impossible’. In four randomly selected patients the catheter was visualized by fluoroscopy after injection of 1.0 ml contrast agent, Iopamiro 300. Three 10 ml volumes of a 1:2 mixture of bupivacaine 0.5% and lidocaine 2% with adrenaline 1:200,000 were slowly—about 5 min total—injected. After the first injection, the second was administered while withdrawing the catheter 2 cm, and the third while withdrawing the catheter another 2 cm. Next the catheter was removed. Patients were returned into supine position. After 25–30 minutes anaesthetic effect and sensory blockade were assessed *once* using pin-prick and the patients were moved into the operating room. Before start of surgery we administered a single IV bolus of 0.5 mg/kg ketamine followed by continuous target-controlled infusion (TCI) of propofol aiming at effect site concentration (Ce) of 1–1.5 mcg/mL to allow spontaneous breathing. Should skin incision or further surgical approach not been tolerated, the regional block would be graded insufficient followed by switch to general anaesthesia.

### Data collection and systemic pain treatment

Patients’ biometric and outcome data (complications) were recorded as well as details of the clinical course, such as amount of anaesthetics used, operation time, surgical difficulties, hemodynamics and oxygenation. At postoperative care unit (PACU) the pain score was assessed at rest and on movement of the shoulder every 15 minutes until 60 minutes using visual rating scale (VRS) from 0 (no pain) to 10 (worst imaginable pain). All patients received one tablet of Ultracet (tramadol plus acetaminophen) and Celebrex 200 mg twice a day. Additional analgesia was provided on demand by IV morphine 0.04 mg/kg boluses with a 15-min dosing interval to maintain VRS—score < 3. Time to first analgesic demand and 24-hr analgesics consumption were recorded. Postoperative nausea and vomiting (PONV) was assessed using a 3-point scale, 0 = no nausea, no vomiting; 1 = nausea, no vomiting; 2 = vomiting with or without nausea. Ondansetron 0.15 mg/kg was administered to patients with PONV score ≥ 1. Complications recorded included Horner’s syndrome, epidural spreading, hypotension (25% reduced from baseline), hypoxia (oxygen saturation < 96%) and adverse effects of local anaesthetics. Patients were checked at 6, 12, and 24 hours postoperatively for adverse effects, pain score, and satisfaction score (1 = poor, 2 = fair, 3 = good, and 4 = excellent). Residents and nurses on PACU and surgical ward both were blinded to the treatment groups. Readiness for discharge was determined by an experienced nurse on the ward, using the Post Anaesthetic Discharge Scoring System (PADSS) [14], notwithstanding that a fixed 2-day surgical discharge protocol was applied. The PADSS scoring includes vital signs, mental status, PONV, bleeding and patients’ intake/output.

### Statistics

Parametric data are expressed as mean ± SD, and non-parametric data as median (range). Intervals, such as duration of surgery, duration of anaesthesia, and PACU time were tested using Mann-Whitney U test. Normality of distributions was tested using the Shapiro-Wilk test. In case of lacking normal distribution data were expressed as median (range). Data were then analyzed using Friedman test. The Mann-Whitney U test was used to analyze differences between the study groups. For post-hoc analysis Wilcoxon’s signed rank tests was used.

Repeated measures analysis of variance were used to compare the groups regarding visual rating score (VRS) over the entire time of evaluation period. The relation between anaesthetic techniques and incidence of complications was evaluated by chi-square or Fisher's exact test as appropriate. Statistical analyses were performed using SPSS v20.0 for Windows (SPSS Inc., Chicago, IL, USA). All tests are two-sided and a p-value of < 0.05 was accepted as statistically significant.

To calculate the sample size the number of patients requiring postoperative morphine in addition to routinely given NSAID was used. The study best comparable to ours was published by Push et al. [15] comparing single shot paravertebral anesthesia with general anesthesia in patients with unilateral breast surgery. The authors reported 13 out of 42 patients (31%) with GA required postoperative NSAID plus opioids compared to 0 out of 44 with paravertebral block. With type I error of 0.05 and power 80%, 22 patients per group were needed. To compensate unpredictable drop outs and giving the study more strength 35 patients per group were included.

## Results

**Fig 1:**
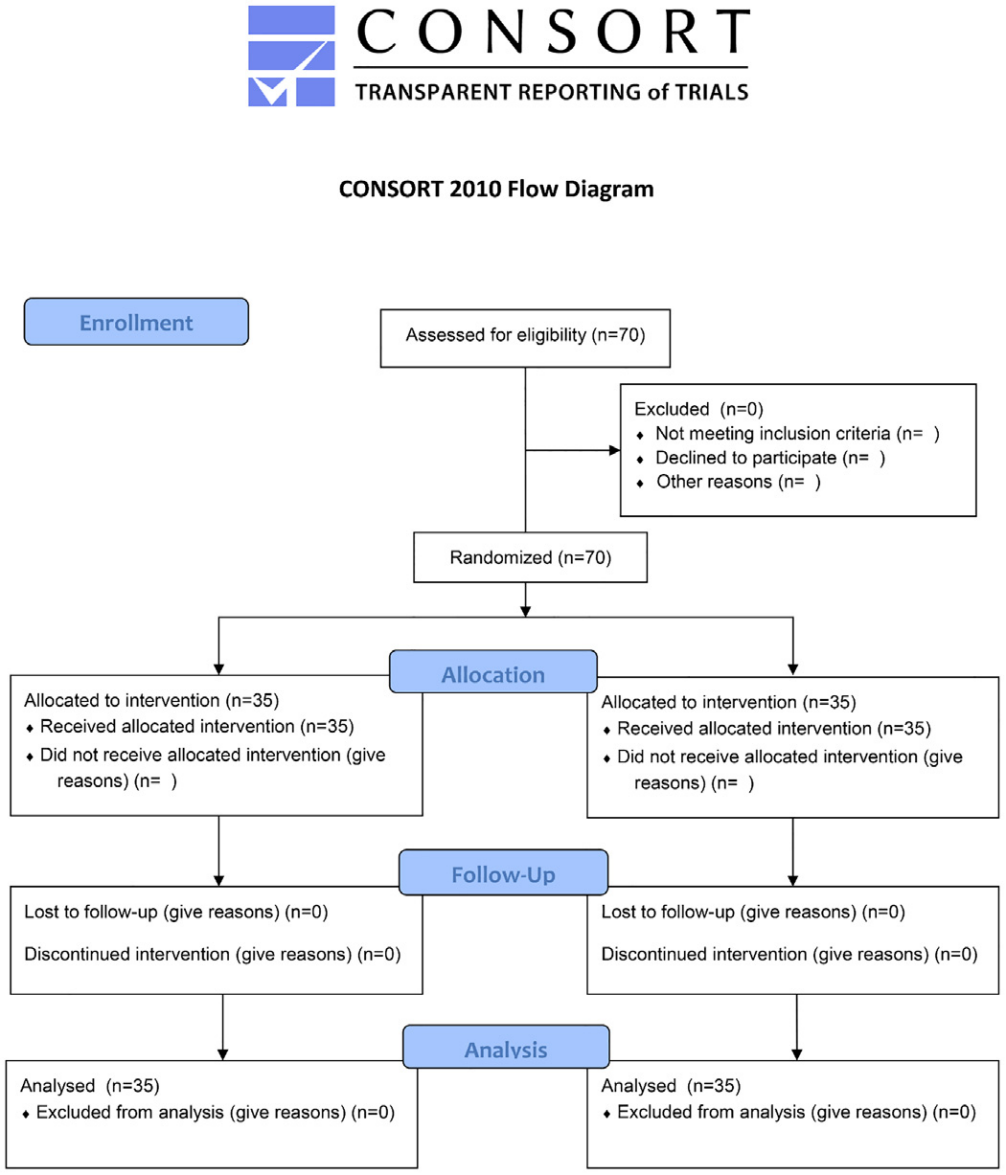
Consort Flow Diagram.

There were no drop outs; all 70 patients enrolled completed the study protocol undergoing unilateral mastectomy surgery with axillary dissection as planned (Fig 1).

All patients with paravertebral block (PVB) had sufficient surgical analgesia, without the need of conversion to general anaesthesia (GA). Perioperatively hemodynamics and oxygenation were stable in all patients. Propofol administration in PVP-patients was 52.6 ± 16.5 mcg x kg^−1^xmin^−1^. There were no complications during the investigation period, neither generally nor related to the respective anaesthetic procedure. Patients’ biometric characteristics and risk scores were similar in both groups (Table 1).

**Table 1:** Demographic data and risk score (ASA—classification)

Characteristics	GA (N = 35)	PVB (N = 35)
Age, years	56.8 ± 9.2	54.0 ± 11.9
BMI, kg/m2	24.7 ± 3.9	24.6 ± 3.7
ASA, N (%)		
I	9 (25.7)	7 (20.0)
II	17 (48.6)	19 (54.3)
III	9 (25.7)	9 (25.7)

GA = general anaesthesia, PVP = paravertebral block; Data are mean ± SD, unless otherwise specified; P values are > 0.27

As demonstrated in Table 2 duration of surgery and PACU time were similar in both groups, whereas anaesthesia time was significantly shorter in GA- compared to PVB-patients with 100 and 120 minutes respectively. Time to readiness for discharge was significantly longer in patients with GA.

**Table 2:** Perioperative time intervals.

Characteristics	GA (N = 35)	PVB (N = 35)	p-value
Duration of surgery, minutes	80 (40–190)	85 (50–145)	0.930
Duration of Anaesthesia, minutes	100 (75–210)	120 (75–180)	0.04
PACU Time, minutes	60 (60–110)	60 (60–85)	0.15
Readiness for discharge* (hrs), mean ± SD	6.5 ± 2.5	4.9 ± 0.5	0.04

GA = general anaesthesia, PVP = paravertebral block

Data are median (max-min), unless otherwise specified; PACU = Postoperative Care Unit; GA = general anaesthesia, PVP = paravertebral block;

*In accordance with Post Anaesthetic Discharge Scoring System (PADSS) [14]

In seven patients with PVB the catheter insertion was rated ‘difficult’ requiring either additional saline solution to dilate the anatomic space (n = 2; 5.7%) or manipulation (5; 14.3%). In four randomly selected PVB-patients with contrast agent application fluoroscopy demonstrated catheters in correct position without kinking or drifts. In all 4 samples the 1^st^ rib and the contrast media at needle insertion site at T4 and all the way up to T1 level could be easily recognised, which was confirmed by an experienced radiologist.

More patients after GA needed postoperative analgesics (Table 3), which was significant on PACU (14 (40%) vs. 4 (11%); p = 0.006) but not on ward (p = 0.229) and just nearly for the entire stay (p = 0.056).

**Table 3:** Patients with postoperative morphine; *entire stay*.

Patients requiring morphine	Group		P—value
	GA (N = 35)	PVB (N = 35)	
**N (%)**	22 (63)	14 (40)	0.056

Data for morphine consumption and VRS on PACU and on ward did not show normal distribution; therefore they are presented as median and range (Tables 4–6). Analgesic consumption was significantly greater in patients with GA (Table 4), and so was the pain score (VRS) at rest and at movement (Tables 5 and 6) at almost any time (PACU and ward). Interval to first analgesic demand was significantly shorter in GA patients compared to PVB with 43.8 ± 59.9 and 56.7 ± 33.8 min respectively (p = 0.043).

**Table 4:** Morphine consumption (mg) at PACU.

	GA (N = 35)	PVB (N = 35)	p-value
15 min	0(0–4)	0(0–2.5)	0.035
30 min	0(0–2.5)	0(0–2)	0.023
45 min	0(0–3)	0(0–2)	0.152
60 min	0(0–3)	0	0.317

Data are median (min-max)

**Table 5:** Pain assessment on PACU using visual rating scale (VRS; 1–10).

	GA (N = 35)	PVB (N = 35)	p-value
**VRS at rest**			
15 min	2(0–10)	0(0–8)	0.016
30 min	2(0–10)	0(0–7)	0.010
45 min	2(0–10)	0(0–10)	0.005
60 min	2(0–10)	0(0–6)	0.006
**VRS at Movement**			
15 min	3(0–10)	0(0–8)	0.006
30 min	3(0–10)	0(0–7)	0.001
45 min	3(0–10)	0(0–10)	0.002
60 min	2(0–10)	0(0–6)	0.003

Data are median (min-max)

**Table 6:** Pain assessment on the ward using visual rating scale (VRS; 1–10).

	GA (N = 35)	PVB (N = 35)	p-value
**VRS at rest**			
Postop 1–6hr	3(0–7)	2(0–6)	0.002
Postop 6–12hr	1(0–7)	0(0–3)	0.017
Postop 12–24hr	0(0–5)	0(0–3)	0.127
**VRS at Movement**			
Postop 1–6hr	4(0–10)	3(0–6)	0.001
Postop 6–12hr	3(0–8)	2(0–5)	0.006
Postop 12–24hr	2(0–7)	1(0–4)	0.055

Data are median (min-max)

Incidence of nausea and vomiting (PONV) was 0–5.7% on PACU with maximum 45 minutes after surgery, and 5.7%- 22.9% on the ward with maximum 1–6 hours postoperatively. Overall incidence and severity of (PONV) was higher after general anaesthesia, but the difference was not significant. Nineteen patients (54.3%) after GA reported sore throat. Patient satisfaction was comparable in both groups rated as ‘very good’ or ‘excellent’.

## Discussion

Paravertebral block (PVB) with catheter as sole anaesthesia was effective in all 35 patients. No patient had to be switched to GA, and no additional analgesics were required intraoperatively after the small single dose of ketamine given prior to surgery. However, even a very small ketamine dose has to be considered as a confounding factor, as patients with general anesthesia did not have any ketamine, but fentanyl for analgesia. The efficiency of PVB in our study suggests that ketamine prior to surgical incision is expendable. The propofol dosage given for continuous sedation did not compromise spontaneous breathing and does not provide analgesia. Compared to general anaesthesia (GA) less patients with paravertebral block needed postoperative pain therapy. Compared to Pusch et al. [15], whose data were used for sample size calculation, additional morphine requirement was markedly different. In Pusch’s study only 31% GA-patients and no (0%) PVB-patient needed morphine additional to NSAID, which was true in 63% and 40% resp. of our patients. However, our patients underwent considerably more extensive surgery, such as axillary approach: 100% in our study and only one patient (1.2%) in the study of Pusch. Patients with PVB had less postoperative analgesics requirement, lower pain scores, longer interval for first pain medication, earlier readiness for discharge and similar PONV rate.

### Catheter position

Applying the described PVB catheter-technique local anaesthetics can be injected at different sites, given that the catheter is deep enough with no kinking. Therefore the proper position of the catheter is crucial. Though the puncture was perpendicular to the skin and the needle was redirected in some but not all patients maximal 10°, which is from 90 to 80°, all catheters could be advanced into proper position. Only in seven patients we either had to manipulate the needle or add fluid to dilate the paravertebral space. We guess the accurate position of the needlepoint is crucial, which can be achieved best using ultrasound. Clinical effect and lack of significant side effects in all PVB patients indicated the catheters being in the scheduled position, which was confirmed by radiography after contrast agent injection in 4 patients, and spread of LA observed with ultrasound during all injections. Ultrasound using the linear-probe technique was applied during the whole process of needle insertion incl. the moment of loss of resistance (LOR), and during the three times of volume injection (10 ml each). Accuracy of ultrasound was excellent; pleura movement during injection could be observed. Usefulness of sonography to control needle insertion depth during PVB application has been demonstrated by Pusch et al. [16] in female patients undergoing unilateral breast surgery. Due to limited tissue penetration of linear probes [17] the complete LA-spreading upward after the first injection at T1 could not be observed with certainty. However, a kinking of the catheters can be excluded with high likelihood.

### Doses of local anaesthetics used

The patients’ received 10 ml Bupivacaine 0.5% (50 mg) plus 20 ml Lidocaine 2% (400 mg). Mixing local anaesthetics therapists try to take advantage from both, fast onset (2% Lidocaine) and long lasting effect (0.5% Bupivacaine). Maximum doses are still debated [18,19], and there are no conclusive data adding this way or that way to the controversy. The opinion of de Jong [20] that mixture of LA doesn’t increase the toxicity of the parent drug was not disproved yet. Recommended maximal doses, when adding epinephrine as we did, are 175–225 mg for Bupivacaine and 500 mg for Lidocaine [21]. We consider the mixture of 50 and 400 mg respectively to be safe.

### PVB properties and practice

Our study is the first with one puncture and three injections at different dermatome levels as sole anaesthetic technique in extensive breast surgery. Using a catheter in proper position enables multiple injections at different sites and at a single puncture. In the few prospective randomized studies so far comparing PVB as sole anaesthetic technique with general anaesthesia for breast surgery, from one up to seven injections (punctures) have been applied [5,8,9,15,22,23], but never with a 100% success rate as in our study. Pusch et al. [15] reported 44 patients with breast surgery receiving single injection PVB at T4 level and a failure rate of 6.8%. Cooter et al. [5] performed 172 single-injections PVB in 100 patients with submuscular breast augmentation; the failure rate was 13%. Naja and co-workers [24] performed PVB with single, two, three or four injections (punctures) at different levels in a mixed patient-population undergoing mainly mastectomy (22–61%) and laparoscopic cholecystectomy (33–52%). Four injections resulted in sufficient anaesthesia in 97% of the patients, whereas one injection was effective in only 11%. Klein et al. [8] applied seven paravertebral injections, 4 ml each (T1–T7), in patients with cosmetic breast surgery, as sole anaesthetic technique with an overall failure rate of 13.3%.

The incidence of relevant complications associated with PVB may be low [10,25]. The severity of events however, such as pleural or vascular puncture, severe hypotension, bilateral epidural and intrathecal spread [2,10,25,26] should be a matter of concern. Thomas et al. reported a case of pulmonary hemorrhage after paravertebral block [13], which was commented by Hill and Greengrass [27]. In their response Thomas et al. stated, ‘We would question the wisdom of multiple injections given that even in experts hand there is a risk of complications with each pass of the needle’, something we completely agree on. Though there are no conclusive data yet including our study, it can be assumed that the complication rate increases with the number of punctures being applied per patient. The method described in this study enables the application of PVB as sole anaesthesia even in extended unilateral surgery, without the risk of multiple punctures.

We did not experience relevant anatomy-related difficulties or clinical complications. However, waiting for the onset of PVB caused a 20-minutes preoperative delay, which may interfere with organisational and financial issues, especially in ambulatory surgery. Twenty minutes are equivalent to 0.33 hours; something we have to bear in mind judging readiness for discharge, thus being 5.23 instead of 4.9 hrs for PVB patients. Additionally it is no method for beginners, thus limiting its use for some departments during daily routine.

Paravertebral block cannot replace epidural analgesia (EDA); we do not see the two methods in competition. We agree with Breivik and Norum [11], that downgrading EDA and upgrading PVB is not justified.

### Strength of the study

Our study demonstrates that using a paravertebral catheter sufficient surgical analgesia for extensive breast surgery is achievable. Most importantly the method allows control of the spread of analgesia. Hence catheter-PVB as described adds to the repertoire of neuroaxial blocks, particularly when unilateral thoracic anaesthesia/analgesia with adequate spreading but limited sympathetic effect is required.

### Weaknesses of the study, prospects

The method applied in our trial needs outstanding ‘craftsmanship’, excellent knowledge of anatomy and skill in handling ultrasound. The sedation applied, though without analgesic effect may have been overcautious; a lighter sedation should suffice. Regarding postoperative pain control our study does not prove key advantages for PVP compared to GA. Though we found statistically significant differences in morphine requirement a difference of 1.2 mg in total morphine consumption is of no clinical relevance. Regarding VRS-score there have been patients with VRS 10 in both groups, though considerably more after general anaesthesia (Tables 5 and 6). As a conclusion the catheter should remain in situ when strong and long lasting postoperative pain is expected.

Future studies should apply catheter PVB to different surgical procedures and try to find the minimal necessary dose of local anaesthetics for the respective surgical approach. In addition the potential of a paravertebral catheter remaining in situ for postoperative pain therapy needs further evaluation.

## Conclusions

Surgical anaesthesia by multiple-level injection one-puncture PVB with catheter can be an alternative to general anaesthesia not only for extensive breast surgery but in various thoracic unilateral surgical settings. As a potential benefit the catheter can remain in situ for postoperative use. Catheter placement in proper position requires skill and experience. The procedure is time-consuming; hence its application has to be substantiated.

## Supporting Information

S1 CONSORT Checklist CONSORT Checklist. (DOC)

S1 File IRB-Proposal. Original in Thai language.
(DOC)

S2 File IRB-Proposal. Translated into English language.
(DOCX)

S3 File Patient information. Original in Thai language.
(DOC)

S4 File Patient information. Translated into English language.
(DOCX)

S5 File Informed consent form. Original in Thai language.
(DOC)

S6 File Informed consent form. Translated into English language.
(DOCX)

## References

[1] Exadaktylos AK, Buggy DJ, Moriarty DC, Mascha E, Sessler DI (2006) Can anesthetic technique for primary breast cancer surgery affect recurrence or metastasis? Anesthesiology 105: 660–664. 00000542-200610000-00008 [pii]. 17006061 10.1097/00000542-200610000-00008 PMC1615712

[2] Schnabel A, Reichl SU, Kranke P, Pogatzki-Zahn EM, Zahn PK (2010) Efficacy and safety of paravertebral blocks in breast surgery: a meta-analysis of randomized controlled trials. Br J Anaesth 105: 842–852. aeq265 [pii]; 10.1093/bja/aeq265 20947592

[3] Coopey SB, Specht MC, Warren L, Smith BL, Winograd JM, Fleischmann K (2013) Use of preoperative paravertebral block decreases length of stay in patients undergoing mastectomy plus immediate reconstruction. Ann Surg Oncol 20: 1282–1286. 10.1245/s10434-012-2678-7 23064793

[4] Andreae MH, Andreae DA (2013) Regional anaesthesia to prevent chronic pain after surgery: a Cochrane systematic review and meta-analysis. Br J Anaesth 111: 711–720. aet213 [pii]; 10.1093/bja/aet213 23811426 PMC3793661

[5] Cooter RD, Rudkin GE, Gardiner SE (2007) Day case breast augmentation under paravertebral blockade: a prospective study of 100 consecutive patients. Aesthetic Plast Surg 31: 666–673. 10.1007/s00266-006-0230-5 17486400

[6] Kundra P, Varadharajan R, Yuvaraj K, Vinayagam S (2013) Comparison of paravertebral and interpleural block in patients undergoing modified radical mastectomy. J Anaesthesiol Clin Pharmacol 29: 459–464. 10.4103/0970-9185.119133;JOACP-29-459 [pii]. 24249981 PMC3819838

[7] Eason MJ, Wyatt R (1979) Paravertebral thoracic block-a reappraisal. Anaesthesia 34: 638–642. 517716 10.1111/j.1365-2044.1979.tb06363.x

[8] Klein SM, Bergh A, Steele SM, Georgiade GS, Greengrass RA (2000) Thoracic paravertebral block for breast surgery. Anesth Analg 90: 1402–1405. 10825328 10.1097/00000539-200006000-00026

[9] Shkol'nik LD, Vasil'ev VI, Soboleva LV (2006) [Multi-injection thoracic paravertebral anesthesia during breast cancer operations]. Anesteziol Reanimatol 80–85. 17061598

[10] Norum HM, Breivik H (2011) Learning from the past for the present: paravertebral blocks for thoracic surgery are not without risk. Eur J Anaesthesiol 28: 544–545. 10.1097/EJA.0b013e328344d953;00003643-201107000-00013 [pii]. 21666545

[11] Breivik H, Norum H (2013) Risks of serious complications after neuraxial blocks: apparent decrease due to guidelines for safe practice? Acta Anaesthesiol Scand 57: 541–544. 10.1111/aas.12121 23574646

[12] Crawley SM (2006) Coexisting harlequin and Horner syndromes after high thoracic paravertebral block. Br J Anaesth 96: 537–538. 96/4/537-a [pii]; 10.1093/bja/ael039 16549627

[13] Thomas PW, Sanders DJ, Berrisford RG (1999) Pulmonary haemorrhage after percutaneous paravertebral block. Br J Anaesth 83: 668–669. 10673891 10.1093/bja/83.4.668

[14] Chung F, Chan VW, Ong D (1995) A post-anesthetic discharge scoring system for home readiness after ambulatory surgery. J Clin Anesth 7: 500–506. 0952-8180(95)00130-A [pii]. 8534468 10.1016/0952-8180(95)00130-a

[15] Pusch F, Freitag H, Weinstabl C, Obwegeser R, Huber E, Wildling E (1999) Single-injection paravertebral block compared to general anaesthesia in breast surgery. Acta Anaesthesiol Scand 43: 770–774. 10456819 10.1034/j.1399-6576.1999.430714.x

[16] Pusch F, Wildling E, Klimscha W, Weinstabl C (2000) Sonographic measurement of needle insertion depth in paravertebral blocks in women. Br J Anaesth 85: 841–843. 11732516 10.1093/bja/85.6.841

[17] Kimmey MB, Martin RW, Silverstein FE (1992) Clinical application of linear ultrasound probes. Endoscopy 24 Suppl 1: 364–369. 10.1055/s-2007-1010501 1633782

[18] Heavner JE (2004) Let's abandon blanket maximum recommended doses of local anesthetics. Reg Anesth Pain Med 29: 524 S1098733904005504 [pii]. 15635509 10.1016/j.rapm.2004.08.019

[19] Reynolds F (2005) Maximum recommended doses of local anesthetics: a constant cause of confusion. Reg Anesth Pain Med 30: 314–316. S1098733905000568 [pii]. 15898045 10.1016/j.rapm.2005.01.006

[20] de Jong RH, Bonin JD (1981) Mixtures of local anesthetics are no more toxic than the parent drugs. Anesthesiology 54: 177–181. 7469099 10.1097/00000542-198103000-00001

[21] Rosenberg PH, Veering BT, Urmey WF (2004) Maximum recommended doses of local anesthetics: a multifactorial concept. Reg Anesth Pain Med 29: 564–575. S1098733904004547 [pii]. 15635516 10.1016/j.rapm.2004.08.003

[22] Naja MZ, Ziade MF, Lonnqvist PA (2003) Nerve-stimulator guided paravertebral blockade vs. general anaesthesia for breast surgery: a prospective randomized trial. Eur J Anaesthesiol 20: 897–903. 14649342 10.1017/s0265021503001443

[23] Das S, Bhattacharya P, Mandal MC, Mukhopadhyay S, Basu SR, Mandol BK (2012) Multiple-injection thoracic paravertebral block as an alternative to general anaesthesia for elective breast surgeries: A randomised controlled trial. Indian J Anaesth 56: 27–33. 10.4103/0019-5049.93340;IJA-56-27 [pii]. 22529416 PMC3327066

[24] Naja ZM, El-Rajab M, Al-Tannir MA, Ziade FM, Tayara K, Younes F, et al (2006) Thoracic paravertebral block: influence of the number of injections. Reg Anesth Pain Med 31: 196–201. S1098-7339(06)00002-2 [pii]; 10.1016/j.rapm.2005.12.004 16701182

[25] Naja Z, Lonnqvist PA (2001) Somatic paravertebral nerve blockade. Incidence of failed block and complications. Anaesthesia 56: 1184–1188. 2084–2 [pii]. 11736777 10.1046/j.1365-2044.2001.02084-2.x

[26] Fagenholz PJ, Bowler GM, Carnochan FM, Walker WS (2012) Systemic local anaesthetic toxicity from continuous thoracic paravertebral block. Br J Anaesth 109: 260–262. aes126 [pii]; 10.1093/bja/aes126 22581806

[27] Hill RP, Greengrass R (2000) Pulmonary haemorrhage after percutaneous paravertebral block. Br J Anaesth 84: 423–424. 10793618 10.1093/oxfordjournals.bja.a013461

