# Supplementary material for: Paravertebral Catheter for Three-Level Injection in Radical Mastectomy: A Randomised Controlled Study
Source: PLoS One. 2015 Jun 9;10(6):e0129539. doi: 10.1371/journal.pone.0129539 (PMC4461276; doi:10.1371/journal.pone.0129539)
Supplement: S1 File — Original in Thai language. (DOC) [file pone.0129539.s002.doc]

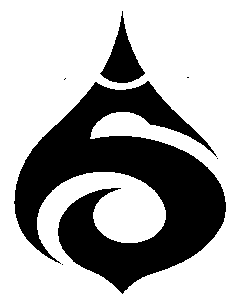
**โครงการวิจัยเพื่อการพิจารณาของคณะกรรมการจริยธรรมการวิจัยในคน**

**คณะแพทยศาสตร์โรงพยาบาลรามาธิบดี**

1. **ชื่อโครงการ**

**ภาษาไทย** การศึกษาเปรียบเทียบประสิทธิภาพของการระงับความรู้สึกด้วย Paravertebral block ชนิดการฉีดยาชาครั้งเดียว และการฉีดยาชาสามระดับผ่านทางสาย (Catheter)

**ภาษาอังกฤษ** Comparison of Single-injection Paravertebral Block (S-PVB) and Three-level Injection Using Paravertebral Catheter (C-PVC) in Breast Surgery

**2. คณะผู้ดำเนินการวิจัย**

**2.1 หัวหน้าโครงการวิจัย**

รศ.พญ.เพชรา สุนทรฐิติ (Assoc.Prof.Petchara Sundarathiti, M.D.)

คุณวุฒิ พบ., วว. วิสัญญีวิทยา

สถานที่ติดต่อได้ ภาควิชาวิสัญญีวิทยา คณะแพทยศาสตร์โรงพยาบาลรามาธิบดี

โทร. 02-2011513, มือถือ 081-8101780

**2.2 ผู้ร่วมวิจัย**

1. Prof.Benno von Bormann, M.D.

Department of Anaesthesiology, Faculty of Medicine Siriraj Hospital

Sayamintr Building 11th floor, 2 Prannok Road, Bangkoknoi, Bangkok, Thailand 10700

E-Mail address: imap@jodu.de

1. ผศ.นพ.รณรัฐ สุวิกะปกรณ์กุล (Ronnarat Suvikapakornkul, M.D.)

คุณวุฒิ พบ., วว. ศัลยศาสตร์

สถานที่ติดต่อได้ ภาควิชาศัลยศาสตร์ คณะแพทยศาสตร์โรงพยาบาลรามาธิบดี

โทร. 02-2011315

1. อ.นพ.ยอดยิ่ง วาสุถิตย์ (Yodying Wasutit, M.D.)

คุณวุฒิ พบ., วว. ศัลยศาสตร์

สถานที่ติดต่อได้ ภาควิชาศัลยศาสตร์ คณะแพทยศาสตร์โรงพยาบาลรามาธิบดี

โทร. 02-2011315

1. รศ.นพ.ภาณุวัฒน์ เลิศสิทธิชัย (Assoc.Prof. Panuwat Lertsithichai, M.D.)

คุณวุฒิ พบ., วว. ศัลยศาสตร์

สถานที่ติดต่อได้ ภาควิชาศัลยศาสตร์ คณะแพทยศาสตร์โรงพยาบาลรามาธิบดี

โทร. 02-2011315

1. อ.พญ.วัลภา อานันทศุภกุล (Vanlapa Arnuntasupakul, M.D.)

คุณวุฒิ พบ., วว. วิสัญญีวิทยา

สถานที่ติดต่อได้ ภาควิชาวิสัญญีวิทยา คณะแพทยศาสตร์โรงพยาบาลรามาธิบดี

โทร. 02-2011513, มือถือ 083-6144136

**3.** **ความสำคัญและที่มาของปัญหา**

**3.1 ทบทวนวรรณกรรม**

จากหลายการศึกษาพบว่าการผ่าตัดเต้านมทำให้เกิดความเจ็บปวดหลังผ่าตัด และภาวะคลื่นไส้อาเจียน รวมทั้งการนำไปสู่ความเจ็บปวดเรื้อรังอย่างมีนัยสำคัญ^1-4^ การระงับความรู้สึกแบบทั่วไปในการผ่าตัดมะเร็งเต้านมพบอุบัติการณ์คลื่นไส้อาเจียนหลังผ่าตัดร้อยละ 50^4^

เมื่อเปรียบเทียบกับการระงับความรู้สึกเฉพาะส่วน ซึ่ง ณ ที่นี้ รวมถึงการระงับความรู้สึกด้านข้างกระดูกสันหลัง (paravertebral block) กับการระงับความรู้สึกแบบทั่วไป พบว่าการระงับความรู้สึกเฉพาะส่วนมีประโยชน์ในการลดปฏิกิริยาความเครียด (stress response) เพิ่มประสิทธิภาพในการระงับปวดและความสามารถในการทำงานของปอดหลังการผ่าตัด ลดโอกาสการกลับเป็นซ้ำของมะเร็งเต้านม และลดภาวะลิ่มเลือดอุดตัน^5-7^ อย่างไรก็ตามผลประโยชน์เหล่านี้ยังมีการศึกษาที่จำกัด จึงยังไม่มีข้อสรุปที่แน่ชัด^8,9^

ปัจจุบัน paravertebral block เพียงอย่างเดียวหรือทำร่วมกับการระงับความรู้สึกโดยทั่วไป ในการผ่าตัดเต้านมเป็นที่นิยมของแพทย์หลายๆ คน เนื่องจากสามารถควบคุมความปวดหลังผ่าตัดได้ดีกว่าและลดระยะเวลานอนโรงพยาบาล^10-13^ อย่างไรก็ตาม จากการศึกษาของ Cheema และคณะ^14^ ได้ทำ paravertebral block ชนิดครั้งเดียว และใช้เครื่องถ่ายภาพรังสีความร้อน (thermographic imaging) หลังฉีด 0.5% bupivacaine 15 มิลลิลิตร ที่ระดับกระดูกสันหลัง T9-10 พบการแพร่กระจายของยาชาจากการฉีดครั้งเดียวนั้น มีค่าเฉลี่ย somatic block 5 ระดับ และ sympathetic block 8 ระดับ โดยชาด้านเดียว ไม่มีผลต่อระบบความดันเลือด คณะผู้ทำการวิจัยไม่เห็นด้วยกับข้อมูลที่ว่า การฉีด paravertebral block ครั้งเดียวนั้นอาจครอบคลุมไม่เพียงพอและบางครั้งอาจเกิดอันตรายได้ แต่อย่างไรก็ตาม การศึกษานี้มีผู้ป่วยเพียง 6 รายที่มีภาวะปวดแบบเรื้อรัง

**3.2 หลักการและเหตุผล**

การฉีด paravertebral block ชนิดครั้งเดียวอาจไม่สามารถระงับความรู้สึกได้ครอบคลุมทุกแนวเส้นประสาทของผิวหนัง (dermatomes) สำหรับการผ่าตัดเต้านมและการผ่าตัดบริเวณรักแร้ (C6-T6) ต้องการการระงับความรู้สึกเฉพาะส่วนที่ครอบคลุมพื้นที่กว้าง (C6-T6) ส่งผลให้ทำการระงับความรู้สึกเฉพาะส่วนแบบฉีดครั้งเดียวอาจจะไม่เพียงพอต่อการผ่าตัด การจากศึกษาของ Naja และคณะ^15^ ได้ทำ bilateral paravertebral block โดยใส่สายฉีดยาจำนวน 1 ครั้ง 2 ครั้ง 3 ครั้ง 4 ครั้ง พบว่าการฉีดยาชาแบบ 4 ครั้งได้ผลดีเพียงพอต่อการผ่าตัด ร้อยละ 97 และการฉีดยาชาแบบ 1 ครั้ง มีประสิทธิภาพดีเพียงร้อยละ 11 สำหรับ Pusch F และคณะ^16^ ได้ทำการศึกษาพบว่าการฉีด paravertebral block แบบครั้งเดียวนั้นเป็นวิธีการที่ง่าย ปลอดภัยและเพียงพอสำหรับการผ่าตัดเต้านม แต่อย่างไรก็ตามวิธีการนี้อาจไม่เพียงพอสำหรับการผ่าตัดที่ต้องครอบคลุมพื้นที่ของการผ่าตัดที่กว้าง ตัวอย่างเช่น การผ่าตัดมะเร็งเต้านมร่วมกับการผ่าตัดบริเวณรักแร้

ถึงแม้กระนั้น การฉีด paravertebral block แบบหลายครั้งอาจเพิ่มความเสี่ยง^17,18^ ดังนั้นการจำกัดจำนวนครั้งของการฉีดยาชาจึงน่าจะปลอดภัยกว่า ซึ่งทำได้โดยการใส่สาย paravertebral ให้อยู่ในตำแหน่งที่เหมาะสม ความลึกเพียงพอและแบ่งฉีดยาชาแบบถอยสายเพื่อให้ยาระงับความรู้สึกกระจายตัวได้อย่างเหมาะสม

**3.3 วัตถุประสงค์งานวิจัย**

เพื่อศึกษาประสิทธิภาพในผู้ป่วยที่เข้ารับการผ่าตัดรักษามะเร็งเต้านม ด้วยการตัดเต้านมแบบข้างเดียว ร่วมกับการผ่าตัดบริเวณรักแร้ด้วยวิธีการระงับความรู้สึกแบบ paravertebral block ชนิดใส่สาย ฉีดยาชาสามครั้งน่าจะได้ปะโยชน์และมีประสิทธิภาพมากกว่าการระงับความรู้สึกแบบทั่วไป

**4. วิธีการวิจัย**

**Double blind, randomized controlled trial**

1. **ผู้เข้าร่วมวิจัย**

ผู้วิจัยใช้การประมาณค่าขนาดของกลุ่มตัวอย่างจากความแตกต่างของความต้องการใช้ opioids+NSAID ของการศึกษาก่อนหน้า^16^ กลุ่ม GA มีความต้องการ opioids+NSAID 31% ในขณะที่กลุ่ม PVB ต้องการ 0% จากจำนวนผู้ป่วยทั้งกลุ่ม เมื่อนำมาคำนวณขนาดตัวอย่างโดยให้ Type I error เป็น 0.05 และ power of test เป็น 80% แล้วจะได้กลุ่มละ 21 คน ผู้วิจัยเผื่อจำนวนเพื่อป้องกัน data loss ดังนั้นสุดท้ายจึงใช้กลุ่มตัวอย่างกลุ่มละ 35 คน

**เกณฑ์การคัดเลือกผู้เข้าร่วมการทำวิจัย (Inclusion criteria)**

- ผู้ป่วยที่เข้ารับการผ่าตัดเต้านมข้างเดียวร่วมกับการผ่าตัดบริเวณรักแร้ที่โรงพยาบาลรามาธิบดี ตั้งแต่ เดือนกันยายน พ.ศ. 2556 ถึง เดือนมกราคม พ.ศ. 2557
- ผู้ป่วยที่มีอายุ 25 – 80 ปี
- ผู้ป่วย ASA physical status I-III
- ผู้ป่วยยินยอมเข้าร่วมโครงการวิจัยด้วยความสมัครใจ และลงชื่อเป็นลายลักษณ์อักษร
  ในใบยินยอมเข้าร่วมการศึกษา (Informed consent form)

**เกณฑ์การคัดเลือกผู้เข้าร่วมการทำวิจัยออกจากโครงการ (Exclusion criteria)**

- ผู้ป่วยปฏิเสธการระงับความรู้สึกวิธี paravertebral block
- มีภาวะติดเชื้อบริเวณที่จะทำการฉีดยาหรือส่วนอื่นของร่างกาย
- มีความผิดปกติของกระดูกไขสันหลังบริเวณอก (thoracic spine)
- มีความผิดปกติของการแข็งตัวของเลือด
- ผู้ป่วยที่มีข้อห้ามการใช้ยา lidocaine, bupivacaine, dormicum, fentanyl, propofol, ketamine ,contrast agent เช่น Iopamiro 300
- ผู้ป่วยโรคอ้วน (obesity, body mass index มากกว่า 30 กิโลกรัม.เซนติเมตร^-2^)
- ผู้ป่วยตั้งครรภ์หรือให้นมบุตร
- ติดตามผลการศึกษาได้น้อยกว่า 24 ชั่วโมง
- ผู้เข้าร่วมการวิจัยปฏิเสธหรือขอถอนตัวจากการวิจัย

**ข. ขั้นตอนและการเก็บข้อมูล**

หลังจากได้รับการอนุมัติจากคณะกรรมการจริยธรรม ผู้วิจัยประเมินผู้ป่วยก่อนได้รับความรู้สึก อธิบายวิธีวิจัยให้ผู้ป่วยเข้าใจและยินยอมลงนามในใบขอรับความยินยอมเข้าร่วมการวิจัย (informed consent) เรียบร้อยแล้ว

ผู้ป่วยจะถูกแบ่งเป็น 2 กลุ่มด้วยวิธี randomization โดยใช้ Block of four method ผู้ป่วย 30 คนได้รับการระงับความรู้สึกแบบทั่วไป (กลุ่ม 1) และผู้ป่วย 30 คนได้รับการระงับความรู้สึกด้วยวิธี paravertebral block ที่ระดับ T4 ชนิดใส่สาย ฉีดยาชาสามครั้ง และผู้ป่วยทุกรายจะได้รับการผ่าตัดโดยศัลยแพทย์ผู้ร่วมวิจัย

เมื่อผู้ป่วยมาถึงห้องรอผ่าตัดติดเครื่องวัดสัญญาณชีพ อันได้แก่ วัดความดันโลหิต, ชีพจร, คลื่นไฟฟ้าหัวใจและ pulse oximetry เรียบร้อยแล้ว

ผู้ป่วยในกลุ่มที่ 1 จะได้การระงับความรู้สึกแบบทั่วไป ด้วยยานำสลบทางหลอดเลือดดำดังนี้ propofol 1-2 mg/kg , fentanyl 1-2 mcg/kg , atracurium 0.5-0.6 mg/kg หลังจากนั้นจึงใส่ท่อช่วยหายใจทางหลอดลม และให้การระงับความรู้สึกต่อด้วย Seveflurane , 50% O_2_ atracurium และfentanyl ตามความเหมาะสม

สำหรับผู้ป่วยกลุ่มที่ 2 จะได้รับการฉีดยา dormicum 0.05 มิลลิกรัมต่อน้ำหนัก1กิโลกรัม ทางหลอดเลือดดำ หลังจากนั้นทำการระงับความรู้สึกโดยวิธี thoracic paravertebral nerve block โดยวิสัญญีแพทย์หัวหน้าโครงงานวิจัยแต่เพียงผู้เดียว ที่ห้องรอผ่าตัดอย่างน้อย 30 นาทีก่อนเริ่มการผ่าตัด ในท่านอนคว่ำ ทำเครื่องหมายที่ตำแหน่งสูงสุดของ spinous process ของระดับ T4 และลงเข็มห่างจากตำแหน่งนั้นมาทางด้านข้าง 2.5 เซนติเมตร หลังจากทำความสะอาดบริเวณนั้นด้วยวิธี aseptic technique แล้ว ใช้เข็ม Tuohy เบอร์ 18 ซึ่งต่อกับ extension tubing และsaline filled syringe ลงเข็มในแนวตั้งฉากกับผิวหนังที่ระดับ T3-4 ให้ปลาย bevel ทำมุมไปด้านหน้า ร่วมกับใช้เครื่องอัลตราซาวน์เพื่อระบุตำแหน่งที่แม่นยำจนปลายเข็มสัมผัสกับ spinous process จากนั้นเปลี่ยนทิศทางเข็มไปในทาง cephalad หรือ caudad และเดินเข็มจนได้ loss of resistance หลังจากนั้นใส่ catheter เบอร์ 20 ผ่านเข็ม Tuohy เบอร์ 18 ที่ตำแหน่ง T3-4 โดยใส่สายลึกประมาณ 8 เซนติเมตรเข้าไปใน paravertebral space โดยผู้ป่วยบางรายอาจได้ฉีด contrast เพื่อดูตำแหน่งของสายด้วย fluoroscope หากห้องผ่าตัดพร้อม และบันทึกผลการใส่เป็น 3 ระดับคือ ใส่ง่าย ใส่ยาก (ต้องขยับเปลี่ยนมุมของเข็มหรือใช้salineเปิดพื้นที่เพิ่มเพื่อให้ใส่สายได้) และไม่สามารถใส่สายได้ หลังจากนั้นทำการ aspiration อย่างระมัดระวังแล้วจึงฉีดยาชาที่ผสมระหว่าง 0.5% bupivacaine 10 มิลลิลิตร และ 2% lidocaine (1:200,000) 20 มิลลิลิตร โดยแบ่งฉีดอย่างช้าๆ ในเวลา 60 วินาทีด้วยปริมาณที่เท่ากัน 3 ตำแหน่งคือ ที่ 8 เซนติเมตร 10 มิลลิลิตร, ที่ 6 เซนติเมตร 10 มิลลิลิตร และที่ 4 เซนติเมตร 10 มิลลิลิตรโดยวิธีการถอยสายทีละ 2 เซนติเมตรหลังจากนั้น paravertebral catheter จะถูกดึงออกอย่างระมัดระวัง

จากนั้นให้ผู้ป่วยอยู่ในท่านอนหงาย แล้วจึงทดสอบระดับการชาโดยใช้ pin-prick technique หลังทดสอบระดับการชาว่าเหมาะสม ให้ผู้ป่วยนอนหงายในท่าพร้อมผ่าตัด เริ่มให้ยา ketamine 0.5 มิลลิกรัม/กิโลกรัม ร่วมกับ propofol โดยเครื่อง Target Controlled Infusion (TCI) ของ Fresenius KABI ตั้งค่า target concentration ที่ 1-1.5 ไมโครกรัมต่อมิลลิลิตร ปรับระดับยาที่ให้ผู้ป่วยหลับ แต่ยังสามารถทำตามคำสั่งได้ เมื่อถูกกระตุ้นได้ (arousable on command) จากนั้นให้ออกซิเจนผ่าน nasal cannula 2-3 ลิตรต่อนาที วัดความดันโลหิตและอัตราการเต้นของหัวใจก่อนเริ่มลงมีดเทียบกับเมื่อเริ่มลงมีดผ่าตัด หากมีค่าสูงขึ้นมากกว่า 25% ของค่าเดิม ให้จัดอยู่ในกลุ่มที่การทำ paravertebral block ไม่เพียงพอต่อการผ่าตัดและให้ยาเพิ่มเติมด้วย ketamine 0.5 มิลลิกรัม/กิโลกรัม ร่วมกับ fentanyl 2-3 ไมโครกรัม/กิโลกรัม ทางหลอดเลือดดำ หากยังไม่เพียงพอต่อการผ่าตัดหรือศัลแพทย์ไม่สามารถผ่าตัดได้ให้เปลี่ยนเป็นการระงับความรู้สึกแบบทั่วไป

หลังการผ่าตัดเสร็จ ผู้ป่วยได้รับการดูแลที่ห้องพักฟื้น โดยพยาบาลที่ห้องพักฟื้นจะไม่ทราบว่าผู้ป่วยได้รับการระงับความรู้สึกในกลุ่มใด จากนั้นจะบันทึกข้อมูลของการใช้ fetanyl ทั้งหมดในห้องผ่าตัดและประเมินระดับความปวดทั้งขณะพักและขณะขยับไหล่ โดยใช้ verbal rating scale (VRS) ที่คะแนนปวด 0 – 10 (0 คือไม่ปวด, 10 คือปวดมากที่สุดเท่าที่จินตนาการได้) ทุก 15 นาทีจนครบ 60 นาที หากมีคะแนนปวดมากกว่า 3 ให้ยา morphine 0.04 มิลลิกรัม/กิโลกรัม ทางหลอดเลือดดำ ทุก 15 นาที จนได้คะแนนความปวดน้อยกว่า 3 คะแนน

สำหรับการประเมินภาวะคลื่นไส้อาเจียนใช้ 3-point scale คือ หากไม่มีภาวะคลื่นไส้อาเจียนให้ 0 คะแนน หากมีภาวะคลื่นไส้แต่ไม่อาเจียนให้ 1 คะแนน และหากมีภาวะอาเจียนแม้จะมีหรือไม่มีภาวะคลื่นไส้ก็ตามให้ 2 คะแนน โดยหากมีคะแนนมากกว่าเท่ากับ 1 คะแนนให้ฉีดยา ondansetron 0.15 มิลลิกรัมต่อกิโลกรัม (ไม่เกิน 8 มิลลิกรัม )

ภาวะแทรกซ้อนต่างๆ ที่เกี่ยวข้องกับการทำหัตการ เช่น Horner’s syndrome การฉีดยาเข้าทาง epidural ความดันต่ำกว่าร้อยละ25 จากเดิม ภาวะมีลมในเยื่อหุ้มปอด และยาชาเป็นพิษจะได้รับการบันทึก

เมื่อผู้ป่วยกลับหออภิบาลจะได้รับยาดังนี้ Ultracet 1 เม็ด และ Celebrex 200 มิลลิกรัม 1 เม็ดวันละ 2 ครั้ง ร่วมกับ lorazepam 0.5 มิลลิกรัม 1 เม็ดก่อนนอน พยาบาลและแพทย์ประจำบ้านไม่ทราบว่าผู้ป่วยได้รับการระงับความรู้สึกในกลุ่มใดซึ่งมีแนวทางในการประเมินความปวดแบบเดียวกัน จะเป็นผู้ประเมิน และจดบันทึกข้อมูลที่ 6, 12 และ 24 ชั่วโมงหลังการผ่าตัด เกี่ยวกับคะแนนความเจ็บปวดและภาวะคลื่นไส้อาเจียน โดยวิธีเดียวกับที่ห้องพักฟื้น, บันทึกจำนวนยาแก้ปวด (opioid) ที่ใช้ทั้งหมด, ความพึงพอใจของผู้ป่วย (แย่ = 1 คะแนน , พอใช้ = 2 คะแนน , ดี = 3 คะแนน , ดีเยี่ยม = 4 คะแนน) และภาวะแทรกซ้อนของการทำหัตถการ

ก่อนกลับบ้าน ให้ผู้ป่วยทำแบบสอบถามคุณภาพการพักฟื้นหลังผ่าตัด (postoperative quality of recovery score ^18^ (the QoR-40)

1. **แบบแผนการวิจัย**

ระยะเวลาดำเนินการวิจัย ประมาณ 6 เดือน คือตั้งแต่ประมาณเดือนพฤศจิกายน 2556 (หรือหลังได้รับการรับรองจริยธรรมฯ) จนถึงเดือน เมษายน 2557

**ตารางการดำเนินการวิจัย**

| **ปี พ.ศ.**  **การดำเนินการ** | **2556** | | | | | | | | **2557** | | | | |
| --- | --- | --- | --- | --- | --- | --- | --- | --- | --- | --- | --- | --- | --- |
|  | พ.ค. | มิ.ย. | ก.ค. | ส.ค. | ก.ย. | ต.ค. | พ.ย. | ธ.ค. | ม.ค. | ก.พ. | มี.ค. | เม.ย. | พ.ค. |
| **ขั้นเตรียมการ** |  |  |  |  |  |  |  |  |  |  |  |  |  |
| - ทบทวนวรรณกรรม | x | x |  |  |  |  |  |  |  |  |  |  |  |
| - เตรียมทีมงานวิจัย | x | x | x |  |  |  |  |  |  |  |  |  |  |
| - ทดสอบระบบจัดเก็บข้อมูล | x | x | x | x | x | x |  |  |  |  |  |  |  |
| - ส่งเอกสารขอรับรองจริยธรรมการวิจัยในคน |  |  |  |  | x | x |  |  |  |  |  |  |  |
| **ขั้นปฏิบัติงาน** |  |  |  |  |  |  |  |  |  |  |  |  |  |
| - คัดเลือกผู้เข้าร่วมการวิจัย |  |  |  |  |  |  | x | x | x | x | x | x |  |
| - Follow-up |  |  |  |  |  |  | x | x | x | x | x | x |  |
| - จัดเก็บและลงข้อมูลในระบบ |  |  |  |  |  |  |  |  |  |  | x | x |  |
| **ขั้นวิเคราะห์ข้อมูล** |  |  |  |  |  |  |  |  |  |  | x | x |  |
| **ขั้นนำเสนอรายงาน** |  |  |  |  |  |  |  |  |  |  |  |  | x |

1. **สถานที่ทำการวิจัย**

ห้องผ่าตัดศัลยกรรม อาคาร 1 โรงพยาบาลรามาธิบดี

1. **อุปกรณ์ที่ใช้**

เข็ม Tuohy เบอร์ 18

เครื่องอัลตราซาวน์

เครื่อง Target Controlled Infusion (TCI)

1. **ความเสี่ยงหรือความไม่สบายที่คาดว่าจะเกิดขึ้นกับผู้ร่วมวิจัย**

**ความเสี่ยง:** การไม่ประสบความสำเร็จของการทำหัตถการ

**การป้องกันและแก้ไข:** ระงับความรู้สึกชนิด paravertebral block ภายใต้วิสัญญีแพทย์ผู้เชี่ยวชาญและมีเครื่องอัลตราซาวด์ชี้นำระหว่างการระงับความรู้สึกทดสอบระดับชาก่อนผ่าตัด ถ้าไม่เพียงพออาจฉีดยาชาเฉพาะที่เพิ่ม หรือให้ยาระงับความรู้สึกทางหลอดเลือดดำเพิ่ม หรือเปลี่ยนเป็นวิธีการระงับความรู้สึกแบบทั่วไป (general anesthesia)

**ความเสี่ยง:** ความดันโลหิตตก หัวใจเต้นช้า

**การแก้ไข:** ให้ยากระตุ้นความดันโลหิต (ephedrine 6-9 มิลลิกรัมต่อครั้ง) และแก้ไขตามสาเหตุ

**ความเสี่ยง:** การแทงทะลุหลอดเลือด, ลมรั่วในเยื่อหุ้มปอด (pneumothorax), เส้นประสาทได้รับความเสียหาย (nerve damage) และภาวะ Horner’s syndrome

**การป้องกัน:** ระงับความรู้สึกชนิด paravertebral nerve blockภายใต้วิสัญญีแพทย์ผู้เชี่ยวชาญและมีเครื่องอัลตราซาวด์ชี้นำระหว่างการระงับความรู้สึก

**ความเสี่ยง:** คลื่นไส้ อาเจียน

**การป้องกัน:** ให้ยาแก้คลื่นไส้ อาเจียน ได้แก่ ondansetron 4 มิลลิกรัม ทางหลอดเลือดดำแก่ผู้ป่วยทุกราย และมีการติดตามอาการข้างเคียงหลังผ่าตัดโดยทีมผู้วิจัย

1. **การวิเคราะห์ข้อมูล (data analysis)**

ข้อมูลแบบพาราเมตริกซ์จะนำเสนอโดย mean±SD และวิเคราะห์เปรียบเทียบสองกลุ่มโดย Student's T test ส่วนข้อมูลแบบนอนพาราเมตริกซ์จะนำเสนอโดย median (range) และวิเคราะห์โดย Mann-Whitney U Test การเปรียบเทียบ VAS ระหว่างกลุ่มใช้ Repeated measures ANOVA การทดสอบความแตกต่างของ incidence of complications ใช้ chi-square หรือ Fisher's exact test การวิเคราะห์ทางสถิติทั้งหมดใช้โปรแกรม SPSS 20.0 (SPSS Inc., Chicago, IL, USA) โดยใช้การทดสอบแบบสองทาง และใช้ค่านัยสำคัญทางสถิติ 0.05

1. **ประโยชน์ที่คาดว่าจะได้รับ**

ใช้เป็นแนวทางการเลือกวิธีระงับความรู้สึกในผู้ป่วยที่เข้ารับการผ่าตัดรักษามะเร็งเต้านมด้วยการตัดเต้านมแบบข้างเดียว ร่วมกับการผ่าตัดบริเวณรักแร้

1. **ข้อพิจารณาด้านจริยธรรม**

การทำวิจัยครั้งนี้ ดำเนินการตามหลักจริยธรรมตามคำประกาศเฮลซิงกิ โดยคำนึงถึงความปลอดภัยของผู้เข้าร่วมงานวิจัยเป็นสำคัญ ทั้งนี้ผู้เข้าร่วมวิจัยได้รับรู้วิธีการวิจัย ตลอดจนผลประโยชน์และผลข้างเคียงที่อาจจะเกิดขึ้นได้ตาม ความเป็นจริงและลงนามในหนังสือยินยอม (informed consent form) โดยแพทย์ได้ดูแลผู้เข้าร่วมการวิจัยทั้งด้านจิตใจและร่างกาย ตลอดจนเตรียมการรักษาในกรณีเกิดภาวะแทรกซ้อน ผู้เข้าร่วมงานวิจัยจะได้รับการดูแลรักษาอย่างถูกต้องตาม หลักวิชาการแพทย์ ไม่ว่าจะยินยอมเข้าร่วมการศึกษาหรือไม่ก็ตาม ผู้เข้าร่วมวิจัยจะสามารถตัดสินใจเข้าร่วมการวิจัยหรือถอนตัวได้อย่างอิสระในทุกขั้นตอนของการวิจัย และผลการศึกษาวิจัยจะไม่มีผลต่อการดูแลรักษาผู้เข้าร่วมการวิจัยในครั้งนี้

1. **การเก็บข้อมูลเป็นความลับ**

ผลงานวิจัยจะเปิดเผยเฉพาะรูปแบบที่เป็นผลสรุปของการวิจัยและไม่มีผลต่อการรักษาใดๆ ของผู้ป่วย ผลการตรวจและการรักษาของผู้ป่วยแต่ละคนจะถูกเก็บไว้เป็นความลับ

**เอกสารอ้างอิง**

(1) Greengrass R, O'Brien F, Lyerly K et al. Paravertebral block for breast cancer surgery. *Can J Anesth.* 1996;43:858-861.

(2) Bhuvaneswari V, Wig J, Mathew PJ, Singh G. Post-operative pain and analgesic requirements after paravertebral block for mastectomy: A randomized controlled trial of different concentrations of bupivacaine and fentanyl. *Indian J Anaesth.* 2012;56:34-39.

(3) Wattwil M, Thorn SE, Lovqvist A, Wattwil L, Gupta A, Liljegren G. Dexamethasone is as effective as ondansetron for the prevention of postoperative nausea and vomiting following breast surgery. *Acta Anaesthesiol Scand.* 2003;47:823-827.

(4) Chan MT, Chui PT, Ho WS, King WW. Single-dose tropisetron for preventing postoperative nausea and vomiting after breast surgery. *Anesth Analg.* 1998;87:931-935.

(5) Naccache N, Jabbour H, Nasser-Ayoub E, Abou ZH, Naja Z. Regional analgesia and breast cancer surgery. *J Med Liban.* 2009;57:110-114.

(6) Schnabel A, Reichl SU, Kranke P, Pogatzki-Zahn EM, Zahn PK. Efficacy and safety of paravertebral blocks in breast surgery: a meta-analysis of randomized controlled trials. *Br J Anaesth.* 2010;105:842-852.

(7) Tahiri Y, Tran de QH, Bouteaud J et al. General anaesthesia versus thoracic paravertebral block for breast surgery: a meta-analysis. *J Plast Reconstr Aesthet Surg.* 2011;64:1261-1269.

(8) Andreae MH, Andreae DA. Local anaesthetics and regional anaesthesia for preventing chronic pain after surgery. *Cochrane Database Syst Rev.* 2012;10:CD007105.

(9) Aufforth R, Jain J, Morreale J, Baumgarten R, Falk J, Wesen C. Paravertebral blocks in breast cancer surgery: is there a difference in postoperative pain, nausea, and vomiting? *Ann Surg Oncol.* 2012;19:548-552.

(10) Das S, Bhattacharya P, Mandal MC, Mukhopadhyay S, Basu SR, Mandol BK. Multiple-injection thoracic paravertebral block as an alternative to general anaesthesia for elective breast surgeries: A randomised controlled trial. *Indian J Anaesth.* 2012;56:27-33.

(11) Arunakul P, Ruksa A. General anaesthesia with thoracic paravertebral block for modified radical mastectomy. *J Med Assoc Thai.* 2010;93 Suppl 7:S149-S153.

(12) Boughey JC, Goravanchi F, Parris RN et al. Prospective randomized trial of paravertebral block for patients undergoing breast cancer surgery. *Am J Surg.* 2009;198:720-725.

(13) Coopey SB, Specht MC, Warren L, Smith BL, Winograd JM, Fleischmann K. Use of preoperative paravertebral block decreases length of stay in patients undergoing mastectomy plus immediate reconstruction. *Ann Surg Oncol.* 2013;20:1282-1286.

(14) Cheema SP, Ilsley D, Richardson J, Sabanathan S. A thermographic study of paravertebral analgesia. *Anaesthesia.* 1995;50:118-121.

(15) Naja ZM, El-Rajab M, Al-Tannir MA et al. Thoracic paravertebral block: influence of the number of injections. *Reg Anesth Pain Med.* 2006;31:196-201.

(16) Pusch F, Freitag H, Weinstabl C, Obwegeser R, Huber E, Wildling E. Single-injection paravertebral block compared to general anaesthesia in breast surgery. Acta Anaesthesiol Scand. 1999;43(7):770-4.

(17) Norum HM, Breivik H. Learning from the past for the present: paravertebral blocks for thoracic surgery are not without risk. *Eur J Anaesthesiol.* 2011;28:544-545.

(18) Norum HM, Breivik H. Published evidence from randomised trials indicates that pain after thoracotomy is more effectively relieved by thoracic epidural analgesia than by paravertebral blocks. *Eur J Anaesthesiol.* 2013;30:261.

**Datasheet for SjPVB vs SjPVC**

1

2

1

Name HN Age DOB

ASA Problem BW kg Ht cm BMI kg/m^2^

Diagnosis Operation Surgeon Op. Time min

Anesthesiologist Anes. Time min PACU Time min

Pulse BPM BP mmHg SpO2 %

**Technique**

□ Group 1 □Group 2 Anes.Level Sensory level (pinprink) Ease of insertion: 🌕Easy 🌕Difficult 🌕Fail Total propofol mg Total ketamine mg Supplement ketamine mg Total dormicum mg

Total fentanyl mcg

Result: □Adequate □Inadequate (Increase BP, HR > 20%) □Fail

**At PACU**

**VRS** (0-10), Morphine 0.04 mg/kg IVprn for VRS>3, every 15 minute

**PONV** (0= no nausea, no vomiting; 1=nausea present, no vomiting; 2=vomiting present with or without nausea), Ondansetron 0.15 mg/kg IV, prn for PONV score ≥1

| VRS | 15min | 30min | 45min | 60min |
| --- | --- | --- | --- | --- |
| Rest/Movement |  |  |  |  |
| Morphine (mg) | 15min | 30min | 45min | 60min |
|  |  |  |  |  |
| PONV | 15min | 30min | 45min | 60min |
|  |  |  |  |  |

At ward: Total morphine

222

| VRS | Postop 1-6hr | Postop 6-12hr | Postop 12-24hr |
| --- | --- | --- | --- |
| Rest/Movement |  |  |  |
| PONV | Postop 1-6hr | Postop 6-12hr | Postop 12-24hr |
|  |  |  |  |

Other problem: □Vascular puncture □Pneumothorax □Nerve damage □Horner’s/Harlequin’s syndrome

Patient’s satisfaction score □แย่ □พอใช้ □ดี □ดีเยี่ยม

Note

Name HN Age

3

**คุณภาพการพักฟื้นหลังผ่าตัด (postoperative quality of recovery score (the QoR-40))**

**ความรู้สึก**

1. รู้สึกสุขสบาย □ใช่ □ไม่ใช่
2. รู้สึกปกติ □ใช่ □ไม่ใช่
3. รู้สึกควบคุมได้ □ใช่ □ไม่ใช่
4. รู้สึกเหมือนฝันร้าย □ใช่ □ไม่ใช่
5. รู้สึกกังวลใจ □ใช่ □ไม่ใช่
6. รู้สึกโกรธ □ใช่ □ไม่ใช่
7. รู้สึกหดหู่ซึมเศร้า □ใช่ □ไม่ใช่
8. รู้สึกโดดเดี่ยว □ใช่ □ไม่ใช่
9. รู้สึกนอนหลับยาก □ใช่ □ไม่ใช่

**ความสุขสบายทางกายภาพ**

1. หายใจได้สบาย □ใช่ □ไม่ใช่
2. นอนหลับสบาย □ใช่ □ไม่ใช่
3. รับประทานอาหารได้ดี □ใช่ □ไม่ใช่
4. รู้สึกผ่อนคลาย □ใช่ □ไม่ใช่
5. คลื่นไส้ □ใช่ □ไม่ใช่
6. อาเจียน □ใช่ □ไม่ใช่
7. คลื่นไส้เหมือนจะอาเจียน □ใช่ □ไม่ใช่
8. รู้สึกกระสับกระส่าย □ใช่ □ไม่ใช่
9. มีอาการสั่น □ใช่ □ไม่ใช่
10. มีอาการหนาวสั่น □ใช่ □ไม่ใช่
11. รู้สึกหนาว □ใช่ □ไม่ใช่
12. รู้สึกเวียนศีรษะ □ใช่ □ไม่ใช่

**การช่วยเหลือด้านจิตใจ**

1. สามารถสื่อสารกับบุคลากรในโรงพยาบาลได้เมื่อรักษาตัวอยู่ในโรงพยาบาล □ใช่ □ไม่ใช่
2. สามารถสื่อสารกับครอบครัวและเพื่อนได้ □ใช่ □ไม่ใช่

44

1. ได้รับการช่วยเหลือจากแพทย์เมื่ออยู่ในโรงพยาบาล □ใช่ □ไม่ใช่
2. ได้รับการช่วยเหลือจากพยาบาลเมื่ออยู่ในโรงพยาบาล □ใช่ □ไม่ใช่
3. ได้รับการช่วยเหลือจากครอบครัวและเพื่อน □ใช่ □ไม่ใช่
4. สามารถเข้าใจถึงความรู้และข้อแนะนำต่างๆ □ใช่ □ไม่ใช่
5. รู้สึกสับสน □ใช่ □ไม่ใช่

**ความสามารถของร่างกาย**

1. สามารถกลับไปทำงานหรือกิจกรรมที่เคยทำที่บ้านได้ □ใช่ □ไม่ใช่
2. สามารถเขียนหนังสือได้ □ใช่ □ไม่ใช่
3. พูดได้เป็นปกติ □ใช่ □ไม่ใช่
4. สามารถอาบน้ำแปรงฟันหรือโกนหนวดได้ □ใช่ □ไม่ใช่
5. สามารถดูแลภาพลักษณ์ตนเองได้ □ใช่ □ไม่ใช่

**ภาวะปวด**

1. ปวดปานกลาง □ใช่ □ไม่ใช่
2. ปวดมาก □ใช่ □ไม่ใช่
3. ปวดศีรษะ □ใช่ □ไม่ใช่
4. ปวดกล้ามเนื้อ □ใช่ □ไม่ใช่
5. ปวดหลัง □ใช่ □ไม่ใช่
6. เจ็บคอ □ใช่ □ไม่ใช่
7. เจ็บปาก □ใช่ □ไม่ใช่

Myles PS, Weitkamp B, Jones K, Merlick J, Hensen S. Validity and reliability of a postoperative quality of recovery score: QoR-40. British Journal of Anaesthesia 84 (1): 11-15 (2000)
