# Supplementary material for: Paravertebral Catheter for Three-Level Injection in Radical Mastectomy: A Randomised Controlled Study
Source: PLoS One. 2015 Jun 9;10(6):e0129539. doi: 10.1371/journal.pone.0129539 (PMC4461276; doi:10.1371/journal.pone.0129539)
Supplement: S2 File — Translated into English language. (DOCX) [file pone.0129539.s003.docx]

**Document submitted to the Ethical clearance committee on human rights
related to research involving human subjects**


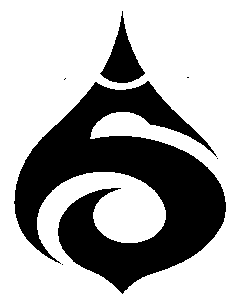


**Faculty of Medicine Ramathibodi Hospital, Mahidol University**

**Translation from Thai to English**

1. **Title** Comparison of Single-injection Paravertebral Block (S-PVB) and Three-level Injection Using Paravertebral Catheter (C-PVC) in Breast Surgery

**2. Research group**

**2.1 Principal investigator**

Dr. Petchara Sundarathiti

Qualification: M.D., Certificate of Anesthesia Fellowship, Royal College of Anesthesiologists of Thailand

Contact: Dept of Anesthesiology, Faculty of Medicine Ramathibodi Hospital,
Mahidol University

Tel: +(66 2) 201 1513, Mobile +(66 8) 1810 1780

**2.2 Co-investigators**

(1) Dr. Benno von Bormann (Corresponding author)

Contact: Dept of Anesthesiology, Faculty of Medicine Siriraj Hospital

Sayamintr Building 11th floor, 2 Prannok Road, Bangkoknoi, Bangkok, Thailand 10700

E-Mail address: imap@jodu.de

(2) Dr. Ronnarat Suvikapakornkul

Qualification: M.D., Certificate of Surgery Fellowship, Royal College of Surgeons of Thailand

Contact: Dept of Surgery, Faculty of Medicine Ramathibodi Hospital,
Mahidol University

Tel: +(66 2) 201 1315

(3) Dr. Yodying Wasutit

Qualification: M.D., Certificate of Surgery Fellowship, Royal College of Surgeons of Thailand

Contact: Dept of Surgery, Faculty of Medicine Ramathibodi Hospital,
Mahidol University

Tel: +(66 2) 201 1315

(4) Dr. Panuwat Lertsithichai

Qualification: M.D., Certificate of Surgery Fellowship, Royal College of Surgeons of Thailand

Contact: Dept of Surgery, Faculty of Medicine Ramathibodi Hospital,
Mahidol University

Tel: +(66 2) 201 1315

(5) Dr. Vanlapa Arnuntasupakul

Qualification: M.D., Certificate of Anesthesia Fellowship, Royal College of Anesthesiologists of Thailand

Contact: Dept of Anesthesiology, Faculty of Medicine Ramathibodi Hospital,
Mahidol University

Tel: +(66 2) 201 1513

**3.** **Introduction**

**3.1 Literature review**

Multiple studies have shown that breast surgery is associated with significant postoperative pain, nausea, vomiting, and development of chronic pain.^(1-4)^ General anaesthesia (GA) is associated with a 50% incidence of nausea and vomiting among breast cancer surgery patients.^(4)^

The proposed benefits of regional anesthesia (RA) including paravertebral block (PVB) compared to GA are decreasing “stress” response, improving postoperative pain control and postoperative pulmonary function, decreasing cancer recurrence, and reducing thromboembolic events.^(5-7)^ However, the debate about these benefits is still controversial.^(8,9)^

PVB for breast surgery, either alone or additional to GA is favored by many investigators, due to better postoperative analgesia and shorter hospital stay.^(10-13)^ In an elder investigation Cheema et al.^(14)^ investigated patients with PVB performed by a single highly experienced therapist using thermographic imaging. After a single injection of 15 ml 0.5% bupivacaine at T9-10, there was a mean distribution of somatic block of five and of sympathetic block of eight dermatomes without any hemodynamic side effects. No bilateral spread was found. The investigators contradicted the suggestions PVB being ineffective and sometimes hazardous. However, they were chronic pain patients with a number of six only.

**3.2 Rational**

Single-injection PVB may not be sufficient to cover all relevant dermatomes for mastectomy with axillary dissection (C6-T6), which is inadequate when applied for surgery as the sole anesthetic technique. The study of Naja and co-workers^(15)^ investigated patients suitable for bilateral PVB. They performed 1, 2, 3 or 4 injections. Four injections resulted in sufficient anaesthesia in 97% of the patients, whereas one injection was effective in only 11%. Pusch et al.^(16)^ found that single-injection PVB for breast surgery was an efficient, simple and safe technique but the study worked on variety of surgical extents. Though there is limited published evidence yet, a single injection for PVB may be insufficient for extensive breast surgery, such as mastectomy including axillary dissection.

However, considering the potential risks of PVB^(17, 18)^ a limitation of injections would be preferable. Inserting a paravertebral catheter (C-PVC) far enough and in proper position offers the opportunity to apply the anesthetic agent at different sites, by moving the catheter backwards after each injection.

**3.3 Objective**

The purpose of our study is to find out if single-injection PVB via C-PVC at three different levels is advantageous and efficient compared to GA in patients having mastectomy with axillary dissection.

**4. Methods**

Double blind, randomized controlled trial

**a. Participants**

The investigators calculated the sample size based on opioids+NSAID requirement from a similar study in a comparable population.^(16)^ It was found that 31% of patients in GA group required opioids+NSAID compared to 0% in PVB group. Thus, to obtain 80% power of test with type I error 0.05, 21 patients in each group is needed. The investigators then add some more in order to prevent data loss during follow-up, so in the end 35 patients a group is desirable.

**Inclusion criteria**

• Patients undergoing unilateral mastectomy surgery with axillary dissection (radical lymph node removal or sentinel node biopsy) from November 2013 to April 2014

• Aged from 25 – 80 years

• ASA physical status I-III

• Written informed consent is obtained

**Exclusion criteria**

• Patients refuse the paravertebral block

• General infection and local infection at the site of planned puncture

• Anatomic deformities of the thoracic spine

• Coagulation disorders

• Allergy against local anesthetics (LA) or contrast agents, such as Iopamiro 300

• Obesity, body mass index more than 30 kg.m^-2^

• Pregnancy or breast-feeding

• Follow-up less than 24 h

• Patients deny or withdraw their consent from the study

**b. Data collection**

After being approved by the Ethics Committee. The investigators evaluate patients before receiving anesthesia. The investigators describe the research methodology and give subjects the sufficient time to consider participation in the study by signing an informed consent.

Subjects are randomly assigned to two groups, 35 in each, by using block of four method. Group I receives GA; Group II receives PVB at level T4 via catheter (C-PVC) with injections at three different levels.

After arrival at the OR, standard monitoring is applied including ECG, noninvasive blood pressure and pulse oxymetry.

Group I: Anesthesia is induced intravenously with propofol 1.5–2 mg/kg and fentanyl 1–2 mcg/kg. Atracurium is used to facilitate tracheal intubation. Anesthesia is maintained with sevoflurane and nitrous oxide in oxygen. Additional Fentanyl 1–2 mcg/kg will be administered on discretion of the anesthesiologist.

Group II: Paravertebral block (PVB) will be entirely performed by one person (1^st^ author) with the patient in prone position. The superior aspect of the T4 spinous process is marked. After skin disinfection an 18-gauge Tuohy needle including attached saline filled syringe is inserted perpendicular to the skin at the T3-4 interspace, using the area 2.5 cm lateral to T4 spinous process as Landmark. Under ultrasound guidance, the needle is advanced by out of plane technique, angled anteriorly until contact with the transverse process, and then redirected either caudal or cephalad and parallel to the spine advanced further until loss of resistance (LOR). Directing the needle parallel to the spine is an alternative to the usual rectangular direction.

After placement of the needle an end-hole 20- gauge catheter is inserted and advanced 8 cm beyond needlepoint into the paravertebral space. The catheter insertion is scored as ‘easy’, ‘difficult’ (manipulation, change of needle direction required, use of saline solution to open the space), or ‘impossible’. In randomly selected patients the catheter localization will be detected by fluoroscopy after injection of 0.5 ml contrast agent. The catheter positioned, a mixture of 10 ml bupivacaine 0.5% plus 20 ml lidocaine 2% with adrenaline 1:200,000 will be slowly injected sequentially 10 ml each while withdrawing the catheter 2 cm after the first and again 2 cm after the second injection. Then the catheter will be removed. Subjects are returned to the supine position and remain for at least 30 minutes in the preoperative area. Sensory blockade is assessed by using pin-prick method.

When analgesia is adequate, subjects are moved into the operating room. Intraoperative sedation is provided with IV ketamine 0.5 mg/kg bolus and continuous propofol using target-controlled infusion (TCI) system aiming at effect site concentration (Ce) of 1-1.5 mcg/mL to allow spontaneous breathing. In case skin incision or further surgical approach is not tolerated, the regional block is graded insufficient, and it is switched to general anesthesia.

1. **Study plan**

The data process will take around 6 months to complete, starting from November 2013 (or after ethical approval) to the end of April 2014.

**Operation schedule**

| **Month/Year**  **Program** | **2013** | | | | | | | | **2014** | | | | |
| --- | --- | --- | --- | --- | --- | --- | --- | --- | --- | --- | --- | --- | --- |
|  | MAY | JUN | JUL | AUG | SEP | OCT | NOV | DEC | JAN | FEB | MAR | APR | MAY |
| **Preparation** |  |  |  |  |  |  |  |  |  |  |  |  |  |
| - Literature review | x | x |  |  |  |  |  |  |  |  |  |  |  |
| - Preparing research team | x | x | x |  |  |  |  |  |  |  |  |  |  |
| - Data collection system testing | x | x | x | x | x | x |  |  |  |  |  |  |  |
| - Ethical approval |  |  |  |  | x | x |  |  |  |  |  |  |  |
| **Operation** |  |  |  |  |  |  |  |  |  |  |  |  |  |
| - Patients’ enrollment |  |  |  |  |  |  | x | x | x | x | x | x |  |
| - Follow-up |  |  |  |  |  |  | x | x | x | x | x | x |  |
| - Data entry |  |  |  |  |  |  |  |  |  |  | x | x |  |
| **Data analysis** |  |  |  |  |  |  |  |  |  |  | x | x |  |
| **Publish the results** |  |  |  |  |  |  |  |  |  |  |  |  | x |

1. **Research setting**

Surgical operating room; 1^st^ building, Ramathibodi Hospital

1. **Equipment**

Touhy needle, size 18

Ultrasound machine

Target Controlled Infusion (TCI) device

1. **Potential risk and/or discomfort that subjects may encounter**

**Risk:** Failure of anesthesia procedure

**Prevention/treatment:** Paravertebral block procedure will be performed under ultrasound guidance by professional anesthesiologists. Sensory blockade is assessed by using pin-prick method. If insufficient anesthesia is suspected, local anesthetic will be injected or adding intravenous sedative or switch to general anesthesia.

**Risk:** Hypotension, bradycardia

**Treatment:** Ephedrine will be administered 6-9 mg per dose and fix the actual cause. **Risk:** Vascular penetration, pneumothorax, nerve damage and Horner’s syndrome

**Prevention:** Paravertebral block procedure will be performed under ultrasound guidance by professional anesthesiologists.

**Risk:** Nausea, vomiting

**Prevention:** Intravenous ondansetron 4 mg to prevent nausea and vomiting and postoperative follow-up for potential adverse effects by investigators

1. **Data analysis**

The parametric data are expressed as mean ± SD and analyzed using Student's T test. The non-parametric data are expressed as median (range) and analyzed using Mann-Whitney U Test. Repeated analysis of variance is used to compare VRS between groups. The association between the techniques and incidence of complications are evaluated by chi-square or Fisher's exact test as appropriate. Statistical analyses will be performed using SPSS v20.0 for Windows (SPSS Inc., Chicago, IL, USA). All tests are two-sided and a p-value of < 0.05 is accepted as statistically significant.

1. **Expected benefits & application**

Guiding factors in selection of anesthesia in patients having mastectomy with axillary dissection

1. **Ethical consideration**

The research is conducted in accordance with the Declaration of Helsinki with regard to the rights and safety of research subjects. All subjects know about the research protocol as well as the benefits and side effects that may occur and have sufficient understanding to sign an informed consent form. The investigator doctors will provide good care for the subjects in both mind and body, and also prepare for the treatments of any complication. All patients will be treated properly according to medical standards whether or not they participate in the study. Subjects will be able to participate or withdraw freely in all phases of the study and the results will not affect the treatment of all subjects.

1. **Confidentiality**

The confidentiality of information supplied by research subjects and the anonymity of respondents is respected.

**References**

(1) Greengrass R, O'Brien F, Lyerly K et al. Paravertebral block for breast cancer surgery. *Can J Anesth.* 1996;43:858-861.

(2) Bhuvaneswari V, Wig J, Mathew PJ, Singh G. Post-operative pain and analgesic requirements after paravertebral block for mastectomy: A randomized controlled trial of different concentrations of bupivacaine and fentanyl. *Indian J Anaesth.* 2012;56:34-39.

(3) Wattwil M, Thorn SE, Lovqvist A, Wattwil L, Gupta A, Liljegren G. Dexamethasone is as effective as ondansetron for the prevention of postoperative nausea and vomiting following breast surgery. *Acta Anaesthesiol Scand.* 2003;47:823-827.

(4) Chan MT, Chui PT, Ho WS, King WW. Single-dose tropisetron for preventing postoperative nausea and vomiting after breast surgery. *Anesth Analg.* 1998;87:931-935.

(5) Naccache N, Jabbour H, Nasser-Ayoub E, Abou ZH, Naja Z. Regional analgesia and breast cancer surgery. *J Med Liban.* 2009;57:110-114.

(6) Schnabel A, Reichl SU, Kranke P, Pogatzki-Zahn EM, Zahn PK. Efficacy and safety of paravertebral blocks in breast surgery: a meta-analysis of randomized controlled trials. *Br J Anaesth.* 2010;105:842-852.

(7) Tahiri Y, Tran de QH, Bouteaud J et al. General anaesthesia versus thoracic paravertebral block for breast surgery: a meta-analysis. *J Plast Reconstr Aesthet Surg.* 2011;64:1261-1269.

(8) Andreae MH, Andreae DA. Local anaesthetics and regional anaesthesia for preventing chronic pain after surgery. *Cochrane Database Syst Rev.* 2012;10:CD007105.

(9) Aufforth R, Jain J, Morreale J, Baumgarten R, Falk J, Wesen C. Paravertebral blocks in breast cancer surgery: is there a difference in postoperative pain, nausea, and vomiting? *Ann Surg Oncol.* 2012;19:548-552.

(10) Das S, Bhattacharya P, Mandal MC, Mukhopadhyay S, Basu SR, Mandol BK. Multiple-injection thoracic paravertebral block as an alternative to general anaesthesia for elective breast surgeries: A randomised controlled trial. *Indian J Anaesth.* 2012;56:27-33.

(11) Arunakul P, Ruksa A. General anaesthesia with thoracic paravertebral block for modified radical mastectomy. *J Med Assoc Thai.* 2010;93 Suppl 7:S149-S153.

(12) Boughey JC, Goravanchi F, Parris RN et al. Prospective randomized trial of paravertebral block for patients undergoing breast cancer surgery. *Am J Surg.* 2009;198:720-725.

(13) Coopey SB, Specht MC, Warren L, Smith BL, Winograd JM, Fleischmann K. Use of preoperative paravertebral block decreases length of stay in patients undergoing mastectomy plus immediate reconstruction. *Ann Surg Oncol.* 2013;20:1282-1286.

(14) Cheema SP, Ilsley D, Richardson J, Sabanathan S. A thermographic study of paravertebral analgesia. *Anaesthesia.* 1995;50:118-121.

(15) Naja ZM, El-Rajab M, Al-Tannir MA et al. Thoracic paravertebral block: influence of the number of injections. *Reg Anesth Pain Med.* 2006;31:196-201.

(16) Pusch F, Freitag H, Weinstabl C, Obwegeser R, Huber E, Wildling E. Single-injection paravertebral block compared to general anaesthesia in breast surgery. Acta Anaesthesiol Scand. 1999;43(7):770-4.

(17) Norum HM, Breivik H. Learning from the past for the present: paravertebral blocks for thoracic surgery are not without risk. *Eur J Anaesthesiol.* 2011;28:544-545.

(18) Norum HM, Breivik H. Published evidence from randomised trials indicates that pain after thoracotomy is more effectively relieved by thoracic epidural analgesia than by paravertebral blocks. *Eur J Anaesthesiol.* 2013;30:261.

**Datasheet for SjPVB vs SjPVC**

Name HN Age DOB

ASA Problem BW kg Ht cm BMI kg/m^2^

Diagnosis Operation Surgeon Op. Time min

Anesthesiologist Anes. Time min PACU Time min

Pulse BPM BP mmHg SpO2 %

**Technique**

□ Group 1 □Group 2 Anes.Level Sensory level (pinprink) Ease of insertion: 🌕Easy 🌕Difficult 🌕Fail Total propofol mg Total ketamine mg Supplement ketamine mg Total dormicum mg

Total fentanyl mcg

Result: □Adequate □Inadequate (Increase BP, HR > 20%) □Fail

**At PACU**

**VRS** (0-10), Morphine 0.04 mg/kg IVprn for VRS>3, every 15 minute

**PONV** (0= no nausea, no vomiting; 1=nausea present, no vomiting; 2=vomiting present with or without nausea), Ondansetron 0.15 mg/kg IV, prn for PONV score ≥1

| VRS | 15min | 30min | 45min | 60min |
| --- | --- | --- | --- | --- |
| Rest/Movement |  |  |  |  |
| Morphine (mg) | 15min | 30min | 45min | 60min |
|  |  |  |  |  |
| PONV | 15min | 30min | 45min | 60min |
|  |  |  |  |  |

At ward: Total morphine

| VRS | Postop 1-6hr | Postop 6-12hr | Postop 12-24hr |
| --- | --- | --- | --- |
| Rest/Movement |  |  |  |
| PONV | Postop 1-6hr | Postop 6-12hr | Postop 12-24hr |
|  |  |  |  |

Other problem: □Vascular puncture □Pneumothorax □Nerve damage □Horner’s/Harlequin’s syndrome

Patient’s satisfaction score □ poor □ fair □ good □ excellent

Note

Name HN Age

**Postoperative quality of recovery score (the QoR-40))**

**Emotional state**

1. Feeling comfortable □Yes □No
2. Having a general feeling of well-being □Yes □No
3. Feeling in control □Yes □No
4. Bad dreams □Yes □No
5. Feeling anxious □Yes □No
6. Feeling angry □Yes □No
7. Feeling depressed □Yes □No
8. Feeling alone □Yes □No
9. Difficulty falling asleep □Yes □No

**Physical comfort**

1. Able to breathe easy □Yes □No
2. Have a good sleep □Yes □No
3. Being able to enjoy food □Yes □No
4. Feeling rested □Yes □No
5. Nausea □Yes □No
6. Vomiting □Yes □No
7. Dry retching □Yes □No
8. Feeling restless □Yes □No
9. Shaking or twiching □Yes □No
10. Shivering □Yes □No
11. Feeling too cold □Yes □No
12. Feeling dizzy □Yes □No

**Psychological support**

1. Able to communicate with hospital staff □Yes □No
2. Able to communicate with family or friends □Yes □No
3. Getting support from hospital doctors (when in hospital) □Yes □No
4. Getting support from hospital nurses (when in hospital) □Yes □No
5. Having support from family or friends □Yes □No
6. Able to understand instructions or advice □Yes □No
7. Feeling confused □Yes □No

**Physical independence**

1. Able to return to work, or usual home activities □Yes □No
2. Able to write □Yes □No
3. Have normal speech □Yes □No
4. Able to wash, brush teeth or shave □Yes □No
5. Able to look after own appearance □Yes □No

**Pain**

1. Moderate pain □Yes □No
2. Severe pain □Yes □No
3. Headache □Yes □No
4. Muscle pains □Yes □No
5. Backache □Yes □No
6. Sore throat □Yes □No
7. Sore mouth □Yes □No

Myles PS, Weitkamp B, Jones K, Merlick J, Hensen S. Validity and reliability of a postoperative quality of recovery score: QoR-40. British Journal of Anaesthesia 84 (1): 11-15 (2000)
