# Supplementary material for: Paravertebral Catheter for Three-Level Injection in Radical Mastectomy: A Randomised Controlled Study
Source: PLoS One. 2015 Jun 9;10(6):e0129539. doi: 10.1371/journal.pone.0129539 (PMC4461276; doi:10.1371/journal.pone.0129539)
Supplement: S3 File — Original in Thai language. (DOC) [file pone.0129539.s004.doc]

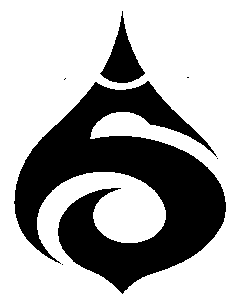
**เอกสารชี้แจงข้อมูล/คำแนะนำแก่ผู้เข้าร่วมการวิจัย**

**(Patient/Participant Information Sheet)**

**ชื่อโครงการ** การศึกษาเปรียบเทียบประสิทธิภาพของการระงับความรู้สึกด้วย Paravertebral block ชนิดการฉีดยาชาครั้งเดียว และการฉีดยาชาสามระดับผ่านทางสาย (Catheter)

**ชื่อผู้วิจัยหลัก** รศ.พญ.เพชรา สุนทรฐิติ (หัวหน้าโครงการวิจัย)

**สถานที่วิจัย**  ภาควิชาวิสัญญีวิทยา คณะแพทยศาสตร์ โรงพยาบาลรามาธิบดี

**บุคคลและวิธีการติดต่อเมื่อมีเหตุฉุกเฉินหรือความผิดปกติที่เกี่ยวข้องกับการวิจัย**

1. รศ.พญ.เพชรา สุนทรฐิติ (Assoc.Prof.Petchara Sundarathiti, M.D.)

­คุณวุฒิ พบ., วว. วิสัญญีวิทยา

สถานที่ติดต่อได้ ภาควิชาวิสัญญีวิทยา คณะแพทยศาสตร์โรงพยาบาลรามาธิบดี

โทร. 02-2011513, มือถือ 081-8101780

1. อ.พญ.วัลภา อานันทศุภกุล (Vanlapa Arnuntasupakul, M.D.)

คุณวุฒิ พบ., วว. วิสัญญีวิทยา

สถานที่ติดต่อได้ ภาควิชาวิสัญญีวิทยา คณะแพทยศาสตร์โรงพยาบาลรามาธิบดี

โทร. 02-2011513, มือถือ 083-6144136

**ผู้สนับสนุนการวิจัย :** ไม่มี

**ความเป็นมาของโครงการ**

มะเร็งเต้านมเป็นมะเร็งที่พบมากที่สุดในเพศหญิง ผู้ป่วยจำนวนมากต้องเข้ารับการผ่าตัดเต้านม โดยร้อยละ 40 ของผู้ป่วยที่ได้รับการผ่าตัดมีภาวะปวดหลังการผ่าตัดจากการรักษาความปวดที่ไม่เพียงพอ ทำให้จำกัดการเคลื่อนไหวหลังผ่าตัด เพิ่มระยะเวลานอนโรงพยาบาล เพิ่มค่ารักษาพยาบาล อีกทั้งยังเพิ่มความเสี่ยงที่จะเปลี่ยนไปเป็นภาวะปวดเรื้อรังอย่างถาวร โดยภาวะปวดเรื้อรังดังกล่าวเกิดได้ถึงร้อยละ 50 และอีกร้อยละ 25 ของผู้ป่วยกลุ่มดังกล่าวมีคุณภาพชีวิตที่ลดลง^1,2,3^ ดังนั้นการวินิจฉัยอย่างรวดเร็วและให้การรักษาที่ดีขึ้นน่าจะเพิ่มประสิทธิภาพในการรักษาผู้ป่วยมะเร็งเต้านมได้มากยิ่งขึ้น

จากหลายการศึกษาพบว่าการผ่าตัดเต้านมพบภาวะปวดและภาวะคลื่นไส้อาเจียนหลังการผ่าตัด รวมถึงการเกิดภาวะปวดเรื้อรัง ซึ่งเกิดได้จากหลายปัจจัย ร้อยละ 50 ของผู้ป่วยที่ได้รับการระงับความรู้สึกแบบทั่วไปในการผ่าตัดรักษามะเร็งเต้านมนั้นมีภาวะคลื่นไส้อาเจียน ดังนั้นการให้การระงับความรู้สึกแบบเฉพาะที่ หรือ การระงับความรู้สึกด้วยการฉีดยาชาเข้าทางด้านข้างของกระดูกสันหลัง (paravertebral block) นั้นจึงมีประโยชน์ในการลดการตอบสนองต่อภาวะเครียด เพิ่มประสิทธิภาพในการระงับปวดและความสามารถในการทำงานของปอดหลังการผ่าตัด ลดการกลับเป็นซ้ำของมะเร็งเต้านม และลดภาวะลิ่มเลือดอุดตัน

**วัตถุประสงค์**

เพื่อศึกษาประสิทธิภาพในผู้ป่วยที่เข้ารับการผ่าตัดรักษามะเร็งเต้านม ด้วยการตัดเต้านมแบบข้างเดียว ร่วมกับการผ่าตัดบริเวณรักแร้ด้วยวิธีการระงับความรู้สึกด้วยการฉีดยาชาเข้าทางด้านข้างของกระดูกสันหลัง (paravertebral block) ที่โรงพยาบาลรามาธิบดี โดยเปรียบเทียบการฉีดยาชาครั้งเดียวแบบใส่สายและการฉีดยาชาครั้งเดียวแบบไม่ใส่สาย รวมถึงการศึกษาความเป็นไปได้ของการใส่สาย ภาวะแทรกซ้อน การระงับปวดหลังผ่าตัด และภาวะคลื่นไส้อาเจียนหลังการผ่าตัด

**รายละเอียดที่จะปฏิบัติต่อผู้เข้าร่วมการวิจัย**

1. หลังจากผ่านการอนุมัติจากคณะกรรมการพิจารณาจริยธรรมการวิจัยในมนุษย์แล้ว คัดเลือกผู้ป่วยที่มารับการระงับความรู้สึกและผ่านตามเกณฑ์การคัดเลือก

2. ผู้วิจัยอธิบายวิธีวิจัยให้ผู้ป่วยและตอบข้อสงสัยจนเข้าใจชัดเจนแล้ว ผู้ป่วยเซ็นต์ใบยินยอมเข้าร่วมโครงการวิจัย

3. ทำการสุ่มผู้ป่วยออกเป็นสองกลุ่มโดยใช้ระบบคอมพิวเตอร์ ได้ผลใส่ซองปิดผนึก

4. ผู้ป่วยทุกคนจะได้รับการประเมินก่อนได้รับการระงับความรู้สึกโดยวิสัญญีแพทย์ ติดเครื่องเฝ้าระวังเพื่อประเมินความดันเลือด อัตราการเต้นของหัวใจ อัตราการหายใจ ความอิ่มตัวของออกซิเจนในเลือด ก่อนทำหัตถการผู้ป่วยทุกรายจะได้รับการฉีดยานอนหลับ (Dormicum) 2-5 มิลลิกรัม ทางหลอดเลือดดำตามแต่ความเหมาะสมของผู้ป่วย

5. ให้การระงับความรู้สึกโดยวิธีฉีดยาชาเข้าทางด้านข้างของกระดูกสันหลัง (paravertebral block) ระดับอก ที่ห้องรอผ่าตัดอย่างน้อย 30 นาทีก่อนเริ่มการผ่าตัด ในท่านอนคว่ำ โดยวิธีการฉีดยาจะถูกแบ่งเป็น 2 กลุ่ม

6. ผู้ป่วยกลุ่มที่ 1 ใส่สายเข้าไปในช่องด้านข้างของกระดูกสันหลังลึกประมาณ 6-8 เซนติเมตรผ่านเข็มเบอร์ 18 จากนั้นแบ่งฉีดยาชา (0.5% bupivacaine 10 มิลลิลิตรและ2% lidocaine (1:200,000) 20 มิลลิลิตร)อย่างช้าๆ ด้วยปริมาณที่เท่ากัน 3 ตำแหน่ง คือ ที่ 6-8 เซนติเมตร 10 มิลลิลิตร จากนั้นถอยสายทีละ 2 เซนติเมตรแล้วฉีดอีกจุดละ 10 มิลลิลิตร รวมเป็น 30 มิลลิลิตร ที่ตำแหน่งสาย ลึก 4-6 เซนติเมตร 10 มิลลิลิตร และที่ 2-4 เซนติเมตร 10 มิลลิลิตร

ผู้ป่วยกลุ่มที่ 2 ได้รับการฉีดยาชาชนิดและปริมาณเดียวกันโดยการฉีดยาอย่างช้าๆ ผ่านเข็ม เบอร์ 18 เพียงครั้งเดียวโดยไม่ใส่สาย

7. ให้ผู้ป่วยอยู่ในท่านอนหงาย แล้วจึงทดสอบระดับการชาโดยใช้เข็มทดสอบ

8. หลังทดสอบระดับการชาว่าเหมาะสมแล้ว ให้ผู้ป่วยนอนหงายในท่าพร้อมผ่าตัด เริ่มให้ยานอนหลับทางหลอดเลือดดำ (ketamine 0.5 mg/kg , propofol TCI ให้ระดับยาในเลือดได้ 0.5-1 ไมโครกรัมต่อเดซิลิตร) ปรับระดับยาที่ให้ผู้ป่วยหายใจเอง หลับ แต่ยังสามารถทำตามคำสั่งได้ เมื่อถูกกระตุ้นได้ ในกรณีที่ผู้ป่วยมีระบบไหลเวียนโลหิตที่เปลี่ยนแปลงต่อการลงมีดผ่าตัด (อัตราการเต้นหัวใจหรือความดันโลหิตเพิ่มขึ้น ร้อยละ 20 ของค่าก่อนลงมีดผ่าตัด) ถือว่าระดับความชา “ไม่เพียงพอ” ต่อการผ่าตัด วิสัญญีแพทย์จะเพิ่มยานอนหลับ (ketamine 0.5 mg/kg และ fentanyl 2-3 mcg/kg) ฉีดทางหลอดเลือดดำ

9. ที่ห้องพักฟื้น แพทย์และพยาบาล ผู้บันทึกไม่ทราบว่าคนไข้เป็นกลุ่มที่ 1 หรือ 2 ก่อนทำการบันทึก การประเมินความปวดทำในขณะอยู่นิ่งและขณะขยับหัวไหล่ข้างที่ทำการผ่าตัด โดยให้คะแนน ระหว่าง 0-10 (0 คือไม่ปวด, 10 คือปวดมากที่สุดเท่าที่จินตนาการได้) ทำการประเมิณทุก 15 นาที จนครบ 60 นาที หากมีคะแนนมากกว่า 3 ให้ยาแก้ปวด (morphine 0.04 mg/kg) ทางหลอดเลือดดำ ประเมินภาวะคลื่นไส้อาเจียนหลังการผ่าตัดโดยให้คะแนน ได้แก่ 0 คือไม่มีภาวะคลื่นไส้อาเจียน, 1 คือมีภาวะคลื่นไส้ ไม่มีอาเจียน, 2 คือมีภาวะอาเจียน อาจมีหรือไม่มีภาวะคลื่นไส้ โดยถ้ามีคะแนนมากกว่าหรือเท่ากับ 1ให้ฉีดยาแก้อาเจียน (ondansetron 0.15mg/kg)\

10. เมื่อผู้ป่วยกลับหออภิบาล ประเมิณผู้ป่วยภายในช่วงเวลาระหว่าง 1-6 ชั่วโมงหลังผ่าตัด, 6-12 ชั่วโมงหลังผ่าตัด, 12-24 ชั่วโมงหลังผ่าตัด ประเมินความเจ็บปวด และภาวะคลื่นไส้อาเจียนวิธีเดียวกับที่ห้องพักฟื้น, บันทึกจำนวนยาแก้ปวด (opioid) ที่ใช้ทั้งหมด, ความพึงพอใจของผู้ป่วย ผู้บันทึกจะไม่ทราบว่าคนไข้เป็นกลุ่มที่ 1 หรือ 2 ก่อนทำการบันทึก

**ประโยชน์และผลข้างเคียงที่จะเกิดแก่ผู้เข้าร่วมการวิจัย**

**ประโยชน์**

ท่านจะได้รับประโยชน์จากการระงับความรู้สึกด้วยการฉีดยาชาเข้าทางด้านข้างของกระดูกสันหลัง (paravertebral block) เพื่อผ่าตัดบริเวณเต้านมและรักแร้ ซึ่งมีผลลดการตอบสนองต่อภาวะเครียด ลดภาวะคลื่นไส้อาเจียน เพิ่มประสิทธิภาพในการระงับปวดและความสามารถในการทำงานของปอดหลังการผ่าตัด ลดการกลับเป็นซ้ำของมะเร็งเต้านม และลดภาวะลิ่มเลือดอุดตัน

**ผลข้างเคียง**

ในการศีกษาครั้งนี้อาจมีความเสี่ยงที่จะเกิดการไม่ประสบความสำเร็จของการทำหัตถการ ความดันโลหิตตก หัวใจเต้นช้า การแทงทะลุหลอดเลือด, ลมรั่วในเยื่อหุ้มปอด, เส้นประสาทได้รับความเสียหาย และภาวะคลื่นไส้ อาเจียน แต่อาการดังกล่าวก็พบน้อยอีกทั้งยังสามารถใช้ยาและวิธีป้องกันหรือรักษาได้ เช่น การทำหัตถการด้วยแพทย์ผู้เชี่ยวชาญ การใช้เครื่องอัลตราซาวด์ชี้นำระหว่างการระงับความรู้สึก และการให้ยาเมื่อเกิดภาวะที่ไม่พึงประสงค์

**การเก็บข้อมูลเป็นความลับ**

ผลงานวิจัยนี้จะเผยแพร่ในวารสารทางวิชาการทางการแพทย์โดยไม่ระบุบุคคลหรือข้อมูลส่วนตัวใดๆมีแต่งานวิจัยทางสถิติซึ่งไม่ส่งผลกระทบต่อผู้เข้าร่วมโครงการ

ถ้าท่านมีปัญหาข้องใจหรือรู้สึกกังวลใจกับการเข้าร่วมในโครงการวิจัยนี้ ท่านสามารถติดต่อกับประธานกรรมการ
จริยธรรมการวิจัยในคน สำนักงานวิจัยคณะฯ อาคารวิจัยและสวัสดิการ คณะแพทยศาสตร์โรงพยาบาลรามาธิบดี
โทร. 02-201-1544
