# Supplementary material for: Paravertebral Catheter for Three-Level Injection in Radical Mastectomy: A Randomised Controlled Study
Source: PLoS One. 2015 Jun 9;10(6):e0129539. doi: 10.1371/journal.pone.0129539 (PMC4461276; doi:10.1371/journal.pone.0129539)
Supplement: S4 File — Translated into English language. (DOCX) [file pone.0129539.s005.docx]

**Patient/Participant Information Sheet**


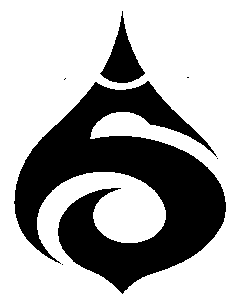


**Translation from Thai to English**

**Project title** Comparison of Single-injection Paravertebral Block (S-PVB) and Three-level Injection Using Paravertebral Catheter (C-PVC) in Breast Surgery

**Principal investigator** Dr. Petchara Sundarathiti

**Institution**  Dept of Anesthesiology, Faculty of Medicine Ramathibodi Hospital

In case of emergency and/or serious problem related to the research please contact

1. Dr. Petchara Sundarathiti

Qualification: M.D., Certificate of Anesthesia Fellowship, Royal College of Anesthesiologists of Thailand

Contact: Dept of Anesthesiology, Faculty of Medicine Ramathibodi Hospital,
Mahidol University

Tel: +(66 2) 201 1513, Mobile +(66 8) 1810 1780

1. Dr. Vanlapa Arnuntasupakul

Qualification: M.D., Certificate of Anesthesia Fellowship, Royal College of Anesthesiologists of Thailand

Contact: Dept of Anesthesiology, Faculty of Medicine Ramathibodi Hospital,
Mahidol University

Tel: +(66 2) 201 1513, Mobile +(66 8) 3614 4136

**Research fund** Not yet

**Introduction**

Multiple studies have shown that breast surgery is associated with significant postoperative pain, nausea, vomiting, and development of chronic pain. General anaesthesia (GA) is associated with a 50% incidence of nausea and vomiting among breast cancer surgery patients.

The proposed benefits of regional anesthesia (RA) including paravertebral block (PVB) compared to GA are decreasing “stress” response, improving postoperative pain control and postoperative pulmonary function, decreasing cancer recurrence, and reducing thromboembolic events. However, the debate about these benefits is still controversial.

PVB for breast surgery, either alone or additional to GA is favored by many investigators, due to better postoperative analgesia and shorter hospital stay. In an elder investigation Cheema et al.investigated patients with PVB performed by a single highly experienced therapist using thermographic imaging. After a single injection of 15 ml 0.5% bupivacaine at T9-10, there was a mean distribution of somatic block of five and of sympathetic block of eight dermatomes without any hemodynamic side effects. No bilateral spread was found. The investigators contradicted the suggestions PVB being ineffective and sometimes hazardous. However, they were chronic pain patients with a number of six only.

Single-injection PVB may not be sufficient to cover all relevant dermatomes for mastectomy with axillary dissection (C6-T6), which is inadequate when applied for surgery as the sole anesthetic technique. The study of Naja and co-workers investigated patients suitable for bilateral PVB. They performed 1, 2, 3 or 4 injections. Four injections resulted in sufficient anaesthesia in 97% of the patients, whereas one injection was effective in only 11%. Pusch et al. found that single-injection PVB for breast surgery was an efficient, simple and safe technique but the study worked on variety of surgical extents. Though there is limited published evidence yet, a single injection for PVB may be insufficient for extensive breast surgery, such as mastectomy including axillary dissection.

However, considering the potential risks of PVB a limitation of injections would be preferable. Inserting a paravertebral catheter (C-PVC) far enough and in proper position offers the opportunity to apply the anesthetic agent at different sites, by moving the catheter backwards after each injection.

**Objective**

The purpose of our study is to find out if single-injection PVB via C-PVC at three different levels is advantageous and efficient compared to GA in patients having mastectomy with axillary dissection.

**Procedures**

1. After being approved by the Ethics Committee. The investigators evaluate patients before receiving anesthesia.

2. The investigators describe the research methodology and give subjects the sufficient time to consider participation in the study by signing an informed consent.

3. Subjects are randomly assigned to two groups, 35 in each, by using block of four method. Group I receives GA; Group II receives PVB at level T4 via catheter (C-PVC) with injections at three different levels.

4. After arrival at the OR, standard monitoring is applied including ECG, noninvasive blood pressure and pulse oxymetry.

Group I: Anesthesia is induced intravenously with propofol 1.5–2 mg/kg and fentanyl 1–2 mcg/kg. Atracurium is used to facilitate tracheal intubation. Anesthesia is maintained with sevoflurane and nitrous oxide in oxygen. Additional Fentanyl 1–2 mcg/kg will be administered on discretion of the anesthesiologist.

Group II: Paravertebral block (PVB) will be entirely performed by one person (1^st^ author) with the patient in prone position. The superior aspect of the T4 spinous process is marked. After skin disinfection an 18-gauge Tuohy needle including attached saline filled syringe is inserted perpendicular to the skin at the T3-4 interspace, using the area 2.5 cm lateral to T4 spinous process as Landmark. Under ultrasound guidance, the needle is advanced by out of plane technique, angled anteriorly until contact with the transverse process, and then redirected either caudal or cephalad and parallel to the spine advanced further until loss of resistance (LOR). Directing the needle parallel to the spine is an alternative to the usual rectangular direction.

5. After placement of the needle an end-hole 20- gauge catheter is inserted and advanced 8 cm beyond needlepoint into the paravertebral space. The catheter insertion is scored as ‘easy’, ‘difficult’ (manipulation, change of needle direction required, use of saline solution to open the space), or ‘impossible’.

6. In randomly selected patients the catheter localization will be detected by fluoroscopy after injection of 0.5 ml contrast agent. The catheter positioned, a mixture of 10 ml bupivacaine 0.5% plus 20 ml lidocaine 2% with adrenaline 1:200,000 will be slowly injected sequentially 10 ml each while withdrawing the catheter 2 cm after the first and again 2 cm after the second injection. Then the catheter will be removed.

7. Subjects are returned to the supine position and remain for at least 30 minutes in the preoperative area. Sensory blockade is assessed by using pin-prick method.

8. When analgesia is adequate, subjects are moved into the operating room.

9. Intraoperative sedation is provided with IV ketamine 0.5 mg/kg bolus and continuous propofol using target-controlled infusion (TCI) system aiming at effect site concentration (Ce) of 1-1.5 mcg/mL to allow spontaneous breathing.

10. In case skin incision or further surgical approach is not tolerated, the regional block is graded insufficient, and it is switched to general anesthesia.

**Benefits and risks**

**Benefits**

Paravertebral block can possibly reduce stress level and the incidence of nausea/vomiting in patients undergoing mastectomy. It also helps relieve postoperative pain, improve lung function, reduce the recurrence of breast cancer and thrombosis.

**Risks**

There may be very few risks of failure in anesthesia procedure, hypotension, bradycardia, vascular penetration, air leak in the pleura, nerves damaged and nausea/vomiting. However, paravertebral block procedure will be performed under ultrasound guidance by professional anesthesiologists. Sensory blockade is assessed by using pin-prick method. If insufficient anesthesia is suspected, local anesthetic will be injected or adding intravenous sedative or switch to general anesthesia.

**Confidentiality**

The confidentiality of information supplied by research subjects and the anonymity of respondents is respected.

If you have questions or feel uncomfortable participating in this research,

Please feel free to contact:

The ethical clearance committee on human rights
related to research involving human subjects

Faculty of Medicine Ramathibodi Hospital, Mahidol University

Tel: +66 2201 1544
