# Supplementary material for: Paravertebral Catheter for Three-Level Injection in Radical Mastectomy: A Randomised Controlled Study
Source: PLoS One. 2015 Jun 9;10(6):e0129539. doi: 10.1371/journal.pone.0129539 (PMC4461276; doi:10.1371/journal.pone.0129539)
Supplement: S5 File — Original in Thai language. (DOC) [file pone.0129539.s006.doc]

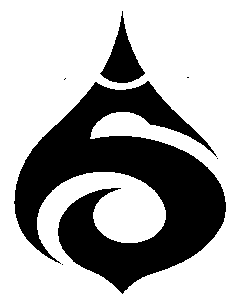
**หนังสือยินยอมโดยได้รับการบอกกล่าวและเต็มใจ**

**(Informed Consent Form)**

ชื่อโครงการ การศึกษาเปรียบเทียบประสิทธิภาพของการระงับความรู้สึกแบบ Paravertebral block โดยใช้การฉีดยาชาสามระดับผ่านทางสาย (Catheter) กับการระงับความรู้สึกแบบทั่วไปในการผ่าตัดเต้านม

ชื่อผู้วิจัยหลัก รศ.พญ.เพชรา สุนทรฐิติ (หัวหน้าโครงการวิจัย)

*ชื่อผู้เข้าร่วมการวิจัย

อายุ เลขที่เวชระเบียน

คำยินยอมของผู้เข้าร่วมการวิจัย

ข้าพเจ้า นาย/นาง/นางสาว ได้ทราบรายละเอียดของโครงการวิจัยตลอดจนประโยชน์ และข้อเสี่ยงที่จะเกิดขึ้นต่อข้าพเจ้าจากผู้วิจัยแล้วอย่างชัดเจน ไม่มีสิ่งใดปิดบังซ่อนเร้นและยินยอมให้ทำการวิจัยในโครงการที่มีชื่อข้างต้น และข้าพเจ้ารู้ว่าถ้ามีปัญหาหรือข้อสงสัยเกิดขึ้นข้าพเจ้าสามารถสอบถามผู้วิจัยได้ และข้าพเจ้าสามารถไม่เข้าร่วมโครงการวิจัยนี้เมื่อใดก็ได้ โดยไม่มีผลกระทบต่อการรักษาที่ข้าพเจ้าพึงได้รับ นอกจากนี้ผู้วิจัยจะเก็บข้อมูลเฉพาะเกี่ยวกับตัวข้าพเจ้าเป็นความลับและจะเปิดเผยได้เฉพาะในรูปที่เป็นสรุปผลการวิจัย การเปิดเผยข้อมูลเกี่ยวกับตัวข้าพเจ้าต่อหน่วยงานต่างๆที่เกี่ยวข้อง กระทำได้เฉพาะกรณีจำเป็นด้วยเหตุผลทางวิชาการเท่านั้น

ลงชื่อ………………………………………(ผู้เข้าร่วมการวิจัย)

………………………………………..(พยาน)

………………………………………..(พยาน)

วันที่ ………………………

**คำอธิบายของแพทย์หรือผู้วิจัย**

ข้าพเจ้าได้อธิบายรายละเอียดของโครงการ ตลอดจนประโยชน์ของการวิจัย รวมทั้งข้อเสี่ยงที่อาจจะเกิดขึ้นแก่ผู้เข้าร่วมการวิจัยทราบแล้วอย่างชัดเจนโดยไม่มีสิ่งใดปิดบังซ่อนเร้น

ลงชื่อ………………………………………(แพทย์หรือผู้วิจัย)

วันที่……………………………

**หมายเหตุ** : กรณีผู้เข้าร่วมการวิจัยไม่สามารถอ่านหนังสือได้ ให้ผู้วิจัยอ่านข้อความในหนังสือยินยอมฯ นี้ให้แก่ผู้เข้าร่วมการวิจัยฟังจนเข้าใจดีแล้ว และให้ผู้เข้าร่วมการวิจัยลงนามหรือพิมพ์ลายนิ้วหัวแม่มือรับทราบในการให้ความยินยอมดังกล่าวข้างต้นไว้ด้วย

* ผู้เข้าร่วมการวิจัย หมายถึง ผู้ยินยอมตนให้ทำวิจัย
