# Supplementary material for: Paravertebral Catheter for Three-Level Injection in Radical Mastectomy: A Randomised Controlled Study
Source: PLoS One. 2015 Jun 9;10(6):e0129539. doi: 10.1371/journal.pone.0129539 (PMC4461276; doi:10.1371/journal.pone.0129539)
Supplement: S6 File — Translated into English language. (DOCX) [file pone.0129539.s007.docx]

**Informed Consent Form**


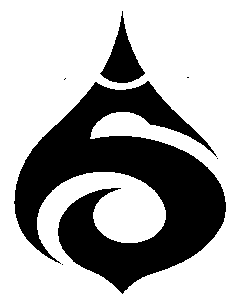


**Translation from Thai to English**

Project title Comparison of Single-injection Paravertebral Block (S-PVB) and Three-level Injection Using Paravertebral Catheter (C-PVC) in Breast Surgery

Principal investigator Dr. Petchara Sundarathiti

*Participant’s name

Age HN

Consent statement

My name is Mr./Mrs./Miss I have known about the research as well as benefits and risks that may occur from the investigators clearly with nothing hidden and I give my consent to the research named above. I know that if I have problems or displeasure, I can withdraw my consent to the research at any time. In addition, the investigator will respect my confidentiality of personal information and reveal only the conclusive research results. Any disclosure of my personal information to the organization involved will be done only if necessary for academic purposes.

Signature………………………………………(Participant)

………………………………………..(Witness 1)

………………………………………..(Witness 2)

Date ………………………

**Investigator’s comment**

I have already explained about the research as well as benefits and risks that may occur clearly with nothing hidden.

Signature………………………………………(Investigator or doctor)

Date……………………………

Note: If the participant is unable to read. The researcher will read the rights and responsibilities statement aloud to the participant prior to obtaining his signature or thumb printed in the consent form.

* Participant, patient who give his/her consent to the research.
